# Global research landscape of inborn errors of immunity: a bibliometric analysis (1991–2025)

**DOI:** 10.1186/s13023-025-04191-4

**Published:** 2026-01-24

**Authors:** Qibin Wu, Jingxian Gao, Yinglin Yuan, Hongji Yang, Qiang Fu

**Affiliations:** 1 Organ Transplant Center, Translational Clinical Immunology key Laboratory of Sichuan Province, Sichuan Provincial People’s Hospital, School of Medicine, University of Electronic Science and Technology of China, No. 32, West Second Section, 1st Ring Road, Qingyang District, Chengdu, 610072 China; 2 Sichuan Jinxin Xinan Women and Children Hospital, Chengdu, China; 3 Department of Gastrointestinal Surgery, Sichuan Provincial People’s Hospital, School of Medicine, University of Electronic Science and Technology of China, Chengdu, China

**Keywords:** Inborn errors of immunity, Gene therapy, Hematopoietic stem cell therapy, Bibliometrics

## Abstract

**Background:**

Inborn errors of immunity (IEI), though individually rare, collectively represent a significant disease burden. From 1980 to 2024, classified IEI disorders expanded from dozens to 559 entities, reflecting advances ranging from immunoglobulin replacement to gene therapy.

**Methods:**

This bibliometric analysis—a comprehensive mapping of the global IEI landscape—analyzed 7,455 publications (1991–2025) from Web of Science Core Collection using Bibliometrix, VOSviewer, and CiteSpace.

**Results:**

Key findings: (1) Annual publication growth: 10.27% (H-index = 173); (2) US dominance: 36.3% publications, 115,221 citations, and TLS = 105,825; (3) Research priorities: Immunodeficiency mechanisms, clinical diagnostics, and key diseases (SCID, CVID, APDS); (4) Therapeutic frontiers: HSCT, gene therapy, targeted signaling inhibitors. (5) Critical gaps: Newborn screening implementation, quality-of-life metrics.

**Conclusion:**

This study provide a comprehensive, multidimensional visualization of the IEI research landscape over 35 years. Although the field maintains a high H-index and broad scope, the pace of research growth appears to have stabilized in the past five years. It is important to note that the observed flattening in total citation counts during this period may be influenced by citation windows and pandemic-related confounding, and should not be interpreted as definitive evidence of field maturity or stagnation. Nonetheless, this observed pattern highlights that that sustaining historical growth rates may require transformative technological advances—particularly in gene editing—to catalyze the next wave of progress in IEI research.

**Supplementary Information:**

The online version contains supplementary material available at 10.1186/s13023-025-04191-4.

## Introduction

Inborn Errors of Immunity (IEI) are a group of genetic disorders caused by monogenic mutations that result in immune system dysregulation [1]. Previously known as Primary Immunodeficiency Diseases (PIDs), the recognized clinical spectrum of IEI has broadened significantly. It now extends beyond the traditional hallmark of recurrent infections to encompass autoimmune manifestations, allergic disorders, inflammatory dysregulation, bone marrow failure, and malignancies [1–3]. This conceptual shift poses greater challenges for clinical diagnosis and management. Furthermore, it positions IEI at the intersection of multiple disciplines, including immunology, genetics, hematology, oncology, and rheumatology, highlighting their increasing research and clinical significance.

The historical trajectory of IEI reflects a deepening understanding of the field, inextricably linked to advances in immunology and genetics. Since Bruton’s seminal 1952 description of X-linked agammaglobulinemia (XLA) [4], the academic paradigm has shifted fundamentally from phenotypic observation to genetic mapping. Early landmark discoveries—including severe combined immunodeficiency (SCID) and chronic granulomatous disease (CGD)—relied primarily on clinical observation and basic immunological assays [5]. Subsequent application of Sanger sequencing and linkage analysis progressively uncovered additional monogenic causes. The 21st-century genomics revolution, driven by next-generation sequencing (NGS), has profoundly transformed IEI research, accelerating the identification of disease-associated genes at an unprecedented pace. Consequently, the International Union of Immunological Societies (IUIS) Expert Committee continually refines the IEI classification system to integrate these discoveries.

The classification of IEI has expanded exponentially, progressing from initial descriptions of dozens of disorders to 559 distinct disease types in the 2024 IUIS update. This classification now encompasses 508 disease-causing genes and 17 phenocopies [1]. Compared to the 2022 schema, this revision adds 67 novel monogenic defects [1, 2], reflecting accelerating discovery. The growth trajectory of IEI classification (1980–2024) [1] visually underscores this expanding nosological landscape. This trend not only mirrors deepening insights into immune system complexity but also highlights the rapid evolution of genetic immunology as a discipline.

In tandem with the deepening understanding of diseases, treatment strategies for IEI have achieved milestone advances, evolving from palliative supportive care toward precise and curative therapies. Immunoglobulin replacement therapy, as the earliest intervention, remains the cornerstone for antibody deficiencies. In 1968, hematopoietic stem cell transplantation (HSCT) was first successfully applied to SCID patients [6], inaugurating immune system reconstitution as a curative approach. Although HSCT achieves remarkable success in severe IEI (2-year overall survival rates up to 90%) [7], its application remains limited by donor availability and complications like graft-versus-host disease (GvHD) [8, 9]. To overcome these limitations, more precise strategies emerged. In 1987, Hershfield et al. demonstrated that polyethylene glycol-modified adenosine deaminase (ADA) enzyme replacement therapy effectively treats ADA-SCID and is superior to erythrocyte infusion [10]. Additionally, precision medicine targeting pathogenic pathways has succeeded in IEI, including Janus kinase (JAK) inhibitors for STAT1/3 gain-of-function mutations [11] and PI3K inhibitors for activated PI3Kδ syndrome (APDS) [12].

The most transformative breakthrough, however, involves gene-level repair. Following the first gene therapy for ADA-SCID in 1993 [13], ex vivo lentiviral hematopoietic stem cell (HSC) gene therapy has demonstrated sustained efficacy and safety in trials. Masiuk et al. confirmed its potential as a curative option for ADA-SCID [14], while a meta-analysis reported high engraftment rates (96.6%; 95% CI: 90.4–98.8%) and low mortality (0.9/100 person-years) [15]. Today, CRISPR/Cas9, base editing, and prime editing technologies are translating “gene repair” into clinical reality [13, 16]. Examples include: The first CRISPR/Cas9-based therapy (Exa-cel) for hemoglobinopathies [17, 18]; The first prime editor therapy (PM359) in CGD trials (NCT06559176) [16]; The first personalized in vivo base-editing treatment [19]; These advances offer ultimate hope for curing IEI patients and accelerate the arrival of individualized gene intervention.

Despite significant advances in IEI—including breakthroughs in molecular mechanisms, refinements in genetic diagnostics, and innovative therapies—research publications have grown exponentially. This proliferation not only reflects intense global scientific focus but also poses a critical challenge: information overload. For researchers, clinicians, and policymakers, this expanding literature makes it increasingly difficult to synthesize the field holistically—whether to comprehend its knowledge architecture, identify key evolutionary milestones, map the global research landscape, or forecast future trends.

While a limited number of bibliometric studies have explored specific subtypes or treatment modalities of IEI—including health-related quality of life (data up to January 2024) [20], HSCT (2013–2022) [21], Wiskott-Aldrich syndrome (2001–2021) [22], and CGD (data up to 2016) [23]—these works are largely confined to narrow subfields. They lack a comprehensive, systematic, and cross-temporal integration of the IEI knowledge ecosystem from a holistic perspective. The lack of this macro-perspective may impede the effective integration of interdisciplinary research, the optimal allocation of scientific research resources, and the accurate prediction of future development directions. Therefore, this study aims to conduct a systematic analysis of 7,455 core documents in the global IEI field from 1991 to 2025 using bibliometric methods. Through quantitative indicators and visualization networks, we will clearly depict the development context, core research forces, the evolution trajectory of research hotspots, and the most promising frontier directions in this field. We hope that this work can provide a valuable “knowledge map” for global scholars, clinicians, and policymakers, thus better guiding future scientific exploration and clinical practice.

## Results

### Annual publication trends

Our systematic search strategy initially identified 10,051 potential articles. Application of predefined screening criteria yielded 7,524 documents, comprising peer-reviewed articles and reviews published exclusively in English. Following exclusion of 69 retracted publications and book chapters, a final corpus of 7,455 (Article 5624; Review 1831) topic-relevant studies was included. This body of work has garnered 221,077 total citations (TC), with 156,046 non-self-citations, and achieves an H-index of 173. These metrics collectively demonstrate the field’s substantial and sustained scholarly influence.

**Fig. 1:**
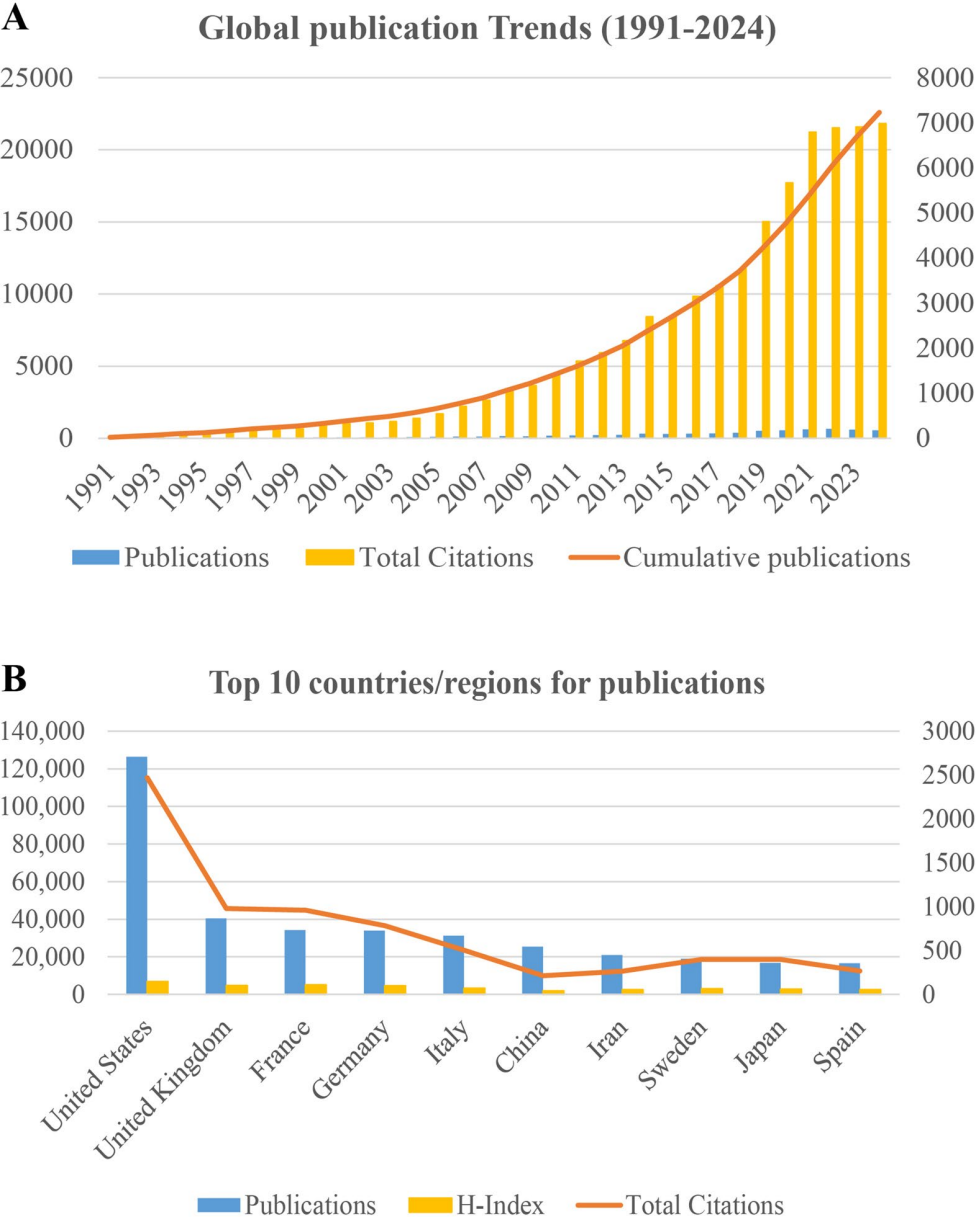
Global publication trends of Inborn error of immunity (1991–2024) and the top 10 countries/regions for publications

The annual publication growth rate averaged 10.27% between 1991 and 2024, with annual output exceeding 500 publications consistently since 2019. Figure 1A illustrates a pronounced upward trajectory in cumulative publications. Notably, TC growth has plateaued since 2021. A marked increase in annual publications occurred around 2020–2021, which coincided with the global COVID‑19 pandemic (Fig. 1A). During this period, several highly cited papers in our dataset examined SARS‑CoV‑2 infection in IEI patients or investigated the role of inborn errors of type I interferon immunity in severe COVID‑19. These observations suggest that the pandemic may have contributed to a short‑term surge in IEI‑related research activity, superimposed on the field’s pre-existing upward trend.

### Country/regional publication trends

The global research landscape encompassed contributions from 128 distinct countries/regions within the 7,455 publications. Figure 1B delineates the top ten productive nations, revealing the preeminent position of the United States with dominant metrics: 36.3% publication share, 115,221 citations, and field-leading H-index (148). The United States sustained an 8.31% annual publication growth rate (1991–2024) with a robust average citation impact of 44 per article (Supplementary Material 1, Figure S1).

Collaboration network analyses further validated this leadership: Total Link Strength (TLS) positioned the United States (105,825) as the central hub, substantially exceeding the United Kingdom (45,586) and France (44,660) (Figure S2). Temporal Production Dynamics confirmed persistent US dominance across decades, followed sequentially by France, UK, Italy, and Germany (Figure S3). Corresponding Authorship Analysis demonstrated US leadership in both single-country (58.7%) and multinational (41.3%) publications (Figure S4). Strategic Partnerships were most frequent with France (327 collaborative works), the UK (288), and Germany (282) (Figure S5).

This convergence of bibliometric indicators—spanning productivity, influence, collaboration centrality, and intellectual stewardship—highlights the United States as the leading contributor to the global IEI research infrastructure. 

### Impact of affiliations and journals

Among 6,244 contributing institutions, Université Paris Cité emerged as the foremost productive organization (1991–2025), as documented in Figure S6. This institution consistently maintains its premier position among the most relevant research entities (Figure S7), reflecting its substantial and sustained contributions to IEI scientific advancement.

Journal analysis identified three dominant publication venues in the field: (1) Journal of Clinical Immunology, (2) Frontiers in Immunology, and (3) Journal of Allergy and Clinical Immunology (Figure S8). These journals demonstrated exceptional scholarly connectivity, securing the top three TLS rankings: (1) Journal of Clinical Immunology (TLS 12,370), (2) Frontiers in Immunology (TLS 9,352), and (3) Journal of Allergy and Clinical Immunology (TLS 8,454) (Figure S9). Notably, Frontiers in Immunology has markedly intensified its engagement with IEI research since 2018, evidenced by accelerating publication volumes in Figures S9 and S10. Complementing these connectivity metrics, local citation analysis revealed the highest-impact journals by H-index: (1) Journal of Allergy and Clinical Immunology (TC = 20,327; H-index = 79), (2) Blood (TC = 19,651; H-index = 56), and (3) Journal of Clinical Immunology (TC = 17,419; H-index = 64) (Figures S11 and S12).

### Author and co-cited author analysis

Figures S13 and S14 identify Jean-Laurent Casanova (214 publications, TC = 6,432) as the most relevant and locally cited author. As shown in Figure S15, Casanova and Steven M. Holland demonstrate the longest continuous IEI research engagement (2001–2025). Table S1 confirms Casanova’s preeminent impact, with leading H-, G-, M-, and TC-indexes. Among 36,085 authors, 161 published ≥ 25 works (Figure S16). Casanova (TLS = 17,791), Hassan Abolhassani (14,326), and Capucine Picard (12,929) ranked highest in TLS.

Analysis of 100,829 co-cited authors revealed 178 cited ≥ 150 times (Figure S17). Stuart G. Tangye, Charlotte Cunningham-Rundles, and Capucine Picard led in TLS and TC among co-cited authors. These metrics collectively demonstrate the scholarly influence and productivity of key contributors to IEI research.

### Analysis of articles and references

**Fig. 2:**
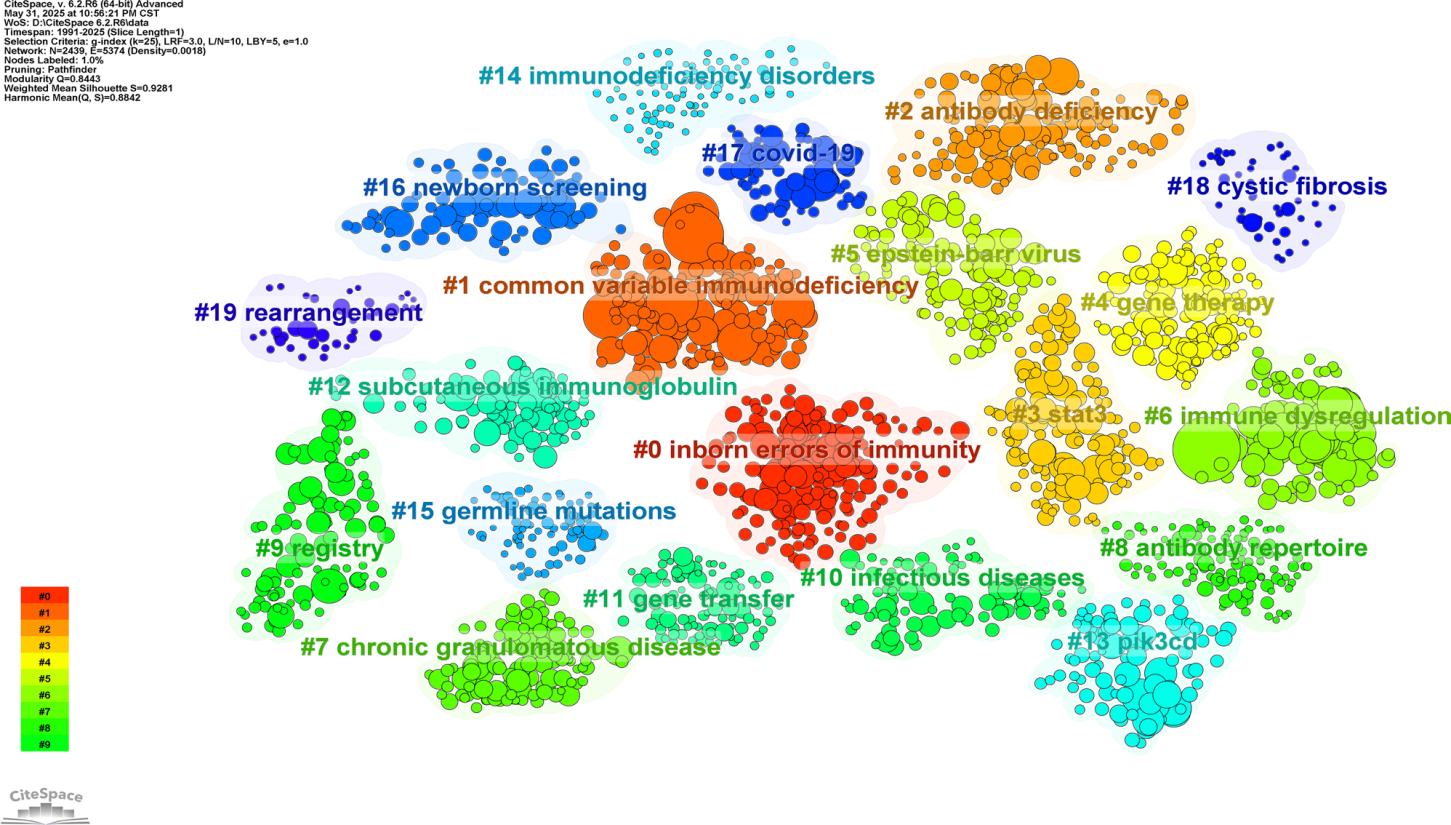
Collaboration network, author, and journal analyses of publications on Inborn error of immunity

Table S2 presents the 20 most highly cited articles, with Mogensen TH’s 2009 Clinical Microbiology Reviews publication (2,205 citations) ranking first. Co-citation network analysis of 159,558 references (Table S3, Figure S18) identified 131 references cited ≥ 100 times. Among these, Tangye SG’s 2020 Journal of Clinical Immunology article had the highest citation count (TC = 512), while Cunningham-Rundles C’s 1999 Clinical Immunology paper demonstrated the strongest scholarly connectivity (TLS = 2,446). Thematic analysis of references revealed 20 distinct research clusters (Fig. 2), with recent scholarship concentrating on six priority domains: (1) Gene defects, (2) Immune dysregulation, (3) STAT proteinopathies, (4) Gene therapy advancements, (5) Infectious disease manifestations, and (6) Newborn screening protocols.

Supplementary Material 2 highlights the 50 references with strongest citation bursts (sustained > 3 years). The 2020 IUIS classification update (Tangye SG, J Clin Immunol 40:24, DOI 10.1007/s10875-019-00737-x) exhibited the most intense burst strength (149.72), reflecting its pivotal role in standardizing IEI nosology.

Notably, the 7/50 references continue to burst into 2025. These publications represent current research priorities and emerging frontiers: (1) Seidel MG et al. [24] advocate clinical diagnosis-guided therapeutic decisions for common variable immunodeficiency (CVID) when genetic diagnoses are unavailable or variants of uncertain significance (VUS) are present; (2) Abolhassani H et al. [25] examine CVID genetic profiles, offering new perspectives on its complex immune phenotypes; (3) Meyts I et al. [26] establish the critical need for severe acute respiratory syndrome coronavirus 2 (SARS-CoV-2) prophylaxis in IEI populations; (4) Zhang Q et al. [27] identify essential type I interferon (IFN) pathway involvement in controlling SARS-CoV-2 through life-threatening coronavirus disease 2019 (COVID-19) case studies, highlighting genetics’ role in disease progression; (5) Bastard P et al. [28] confirm type I IFN’s key function in SARS-CoV-2 protective immunity; (6) Thalhammer J et al. [29] demonstrate that infection-centered diagnostic frameworks miss ∼25% of IEI patients presenting with non-infectious manifestations; (7) Notarangelo LD [5] provides a comprehensive overview of IEI therapeutic advances. Concurrently, a newly published review on IEI (2025) provides a comprehensive synthesis of current insights into both the heightened susceptibility of IEI populations to novel coronavirus infections and the variability in vaccination-induced immune responses observed within these immunocompromised cohorts [30].

### Keyword co-occurrence networks and citation bursts

Figures S19 and S20 present Bibliometrix-derived scientometric analyses of keyword distributions. Figure S19 quantitatively identifies the 100 highest-frequency keywords, with “primary immunodeficiency” predominating at 1,700 occurrences – reflecting the field’s historical nomenclature framework. Figure S20 visualizes longitudinal keyword trajectories (minimum frequency: 10; annual occurrences: ≥3), revealing two significant paradigm shifts: (1) Nosological Transition: The sustained emergence of “inborn errors of immunity” post-2017 directly correlates with the IUIS nomenclature update replacing PID with IEI; (2) Therapeutic Innovation: Co-occurrence of “APDS” and “leniolisib” demonstrates concentrated research investment in molecular pathogenesis and precision therapeutics. These patterns collectively validate the field’s evolution from phenotypic classification toward mechanism-targeted intervention strategies.

**Fig. 3:**
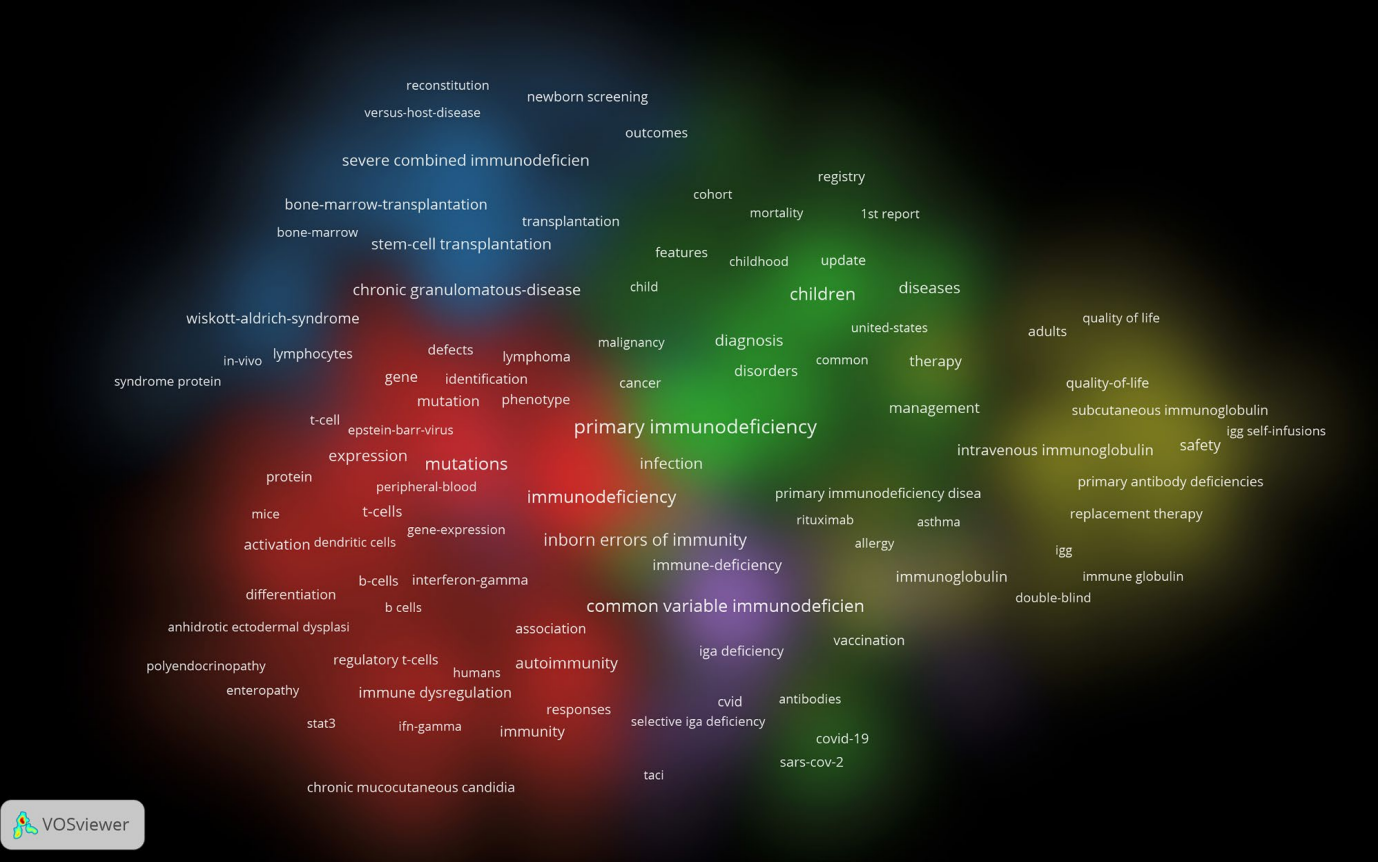
Co-cited author network and thematic analysis of publications on Inborn error of immunity

Employing VOSviewer 1.6.20, we analyzed 17,749 unique keywords extracted from the corpus. Figure 3 visualizes 205 high-occurrence keywords (≥ 50 appearances) clustered into five distinct research domains: (1) Immunobiology (Red Cluster) centers on immunodeficiency mechanisms, incorporating immunocyte pathophysiology (T-cells, B-cells, NK cells, dendritic cells), adaptive immunity, and STAT signaling cascades; (2) Pediatric Epidemiology (Green Cluster) focuses on childhood IEI manifestations, encompassing infectious sequelae, phenotypic spectra (ataxia-telangiectasia, malignancies, asthma, pneumonia), diagnostic frameworks, and population epidemiology (prevalence, mortality, risk stratification); (3) Therapeutic Interventions (Blue Cluster) prioritizes PID treatment modalities, featuring hematopoietic stem cell transplantation (HSCT), gene therapy applications, and disease entities (CGD, SCID, leukocyte adhesion deficiency, Wiskott-Aldrich syndrome, ADA deficiency); (4) Clinical Management (Yellow Cluster) addresses adult care optimization through health-related quality of life metrics, immunoglobulin replacement protocols, and longitudinal treatment strategies; (5) Humoral Deficiencies (Purple Cluster) examines CVID-associated pathologies, including X-linked agammaglobulinemia, Bruton’s tyrosine kinase dysfunction, and hyper-IgM syndromes. This cartography delineates the multidimensional research architecture spanning fundamental mechanisms to clinical management in IEI.

**Fig. 4:**
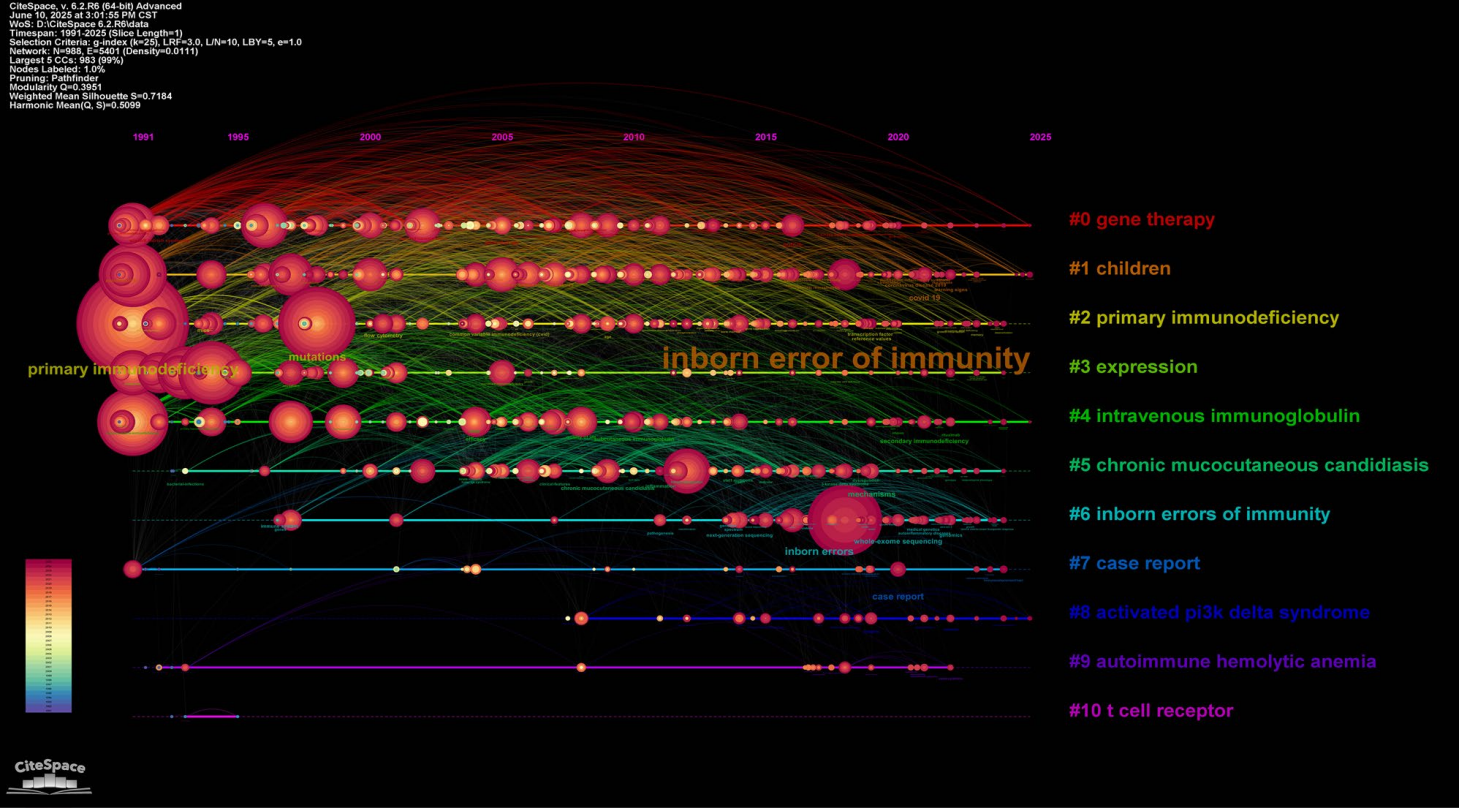
Keyword co-occurrence and network, and timeline analysis

Employing CiteSpace 6.2.R6, we conducted keyword timeline analysis identifying 11 distinct thematic clusters (Fig. 4): gene therapy (cluster #0), children cluster (cluster #1), primary immunodeficiency (cluster #2), expression (cluster #3), intravenous immunoglobulin (cluster #4), chronic mucocutaneous candidiasis (cluster #5), inborn errors of immunity (cluster #6), case report(cluster #7), activated PI3K delta syndrome (cluster #8), autoimmune hemolytic anemia (cluster #9), and T cell receptor (cluster #10). Temporal dynamics reveal significant research evolution: Cluster #10 (T cell receptor) shows diminished activity in recent years. Contemporary research prioritizes: (1) Therapeutic innovations (Cluster #0: Gene therapy); (2) Pediatric manifestations (Cluster #1: Children); (3) Antibody replacement strategies (Cluster #4: IVIg); (4) Targeted pathway disorders (Cluster #8: APDS). Burst intensity mapping (where keyword size correlates with citation burst strength) identifies “inborn errors of immunity” (Cluster #6) as the most prominent burst term within pediatric research. This concentration reflects the disproportionate clinical burden of IEIs in childhood populations. Complementing this, Figure S21 visualizes the top 50 keywords with strongest citation bursts, delineating both sustained research concentrations and emerging domain migrations. This dual-perspective analysis captures the field’s dynamic knowledge progression: (1) Established research pillars (e.g., immunoglobulin therapy); (2) Emerging investigative frontiers (e.g., APDS molecular targeting); (3) Declining thematic interests (e.g., TCR-focused studies); and (4) APDS pathogenesis (Cluster #8).

## Discussion

This study provides a comprehensive bibliometric framework systematically mapping the IEI field’s evolution. Through quantitative analysis of global scholarly output (1991–2025), we delineate: (1) The discipline’s developmental trajectory; (2) Dynamic research hotspot progression; (3) Emerging frontier domains. Key scientometric indicators reveal a field in accelerated transition: (1) Exponential publication growth (>500 annually since 2019); (2) Citation plateau during 2021–2025 despite output expansion. It is important to note that the observed flattening in total citation counts during this period may be influenced by citation windows and pandemic-related confounding. Nevertheless, this divergence still signals both intensifying global research engagement and critical inflection points: (1) Progress indicator: Substantial scholarly focus on IEI pathophysiology and therapeutics; (2) Challenge indicator: Stagnating per-article influence may underscore the urgent need for: conceptual breakthroughs in disease classification, novel therapeutic validation frameworks, and enhanced clinical translation mechanisms. Collectively, these findings position IEI research at a pivotal juncture—demanding integrative strategies to transform quantitative output into qualitative clinical advances. To assess potential database bias, we performed a parallel search in Scopus using identical query terms and date filters (1991–2025). The major findings were consistent with those from Web of Science: publication trends aligned, with a peak in 2022; the top ten productive countries were identical; the article-to-review ratio remained stable at approximately 3:1. Journal sources, national productivity trends, and country collaboration maps also showed substantial congruence. Author-level overlap was high (9/10 among the top 10 most productive authors; 17/20 among the most relevant authors). Institutional overlap was more limited, with only half of the top ten institutions being common to both databases (5/10). The United States remained the leading country for corresponding authors in both single-country and internationally collaborated publications.

A notable surge in publications in 2021 coincided with the COVID-19 pandemic, which amplified research interest in IEIs. This surge was propelled by landmark studies linking inborn errors of type I interferon immunity to severe SARS-CoV-2 infection. Importantly, the upward trajectory of IEI research was an established trend before 2020, fueled by advances in next-generation sequencing, evolving IUIS classifications, and progress in gene and cell therapies. Thus, the pandemic acted more as an accelerator than the origin of this growth. Taken together, these patterns place IEI research at a pivotal juncture, highlighting the imperative to translate rising quantitative output into sustained qualitative and clinical advances.

From a global research architecture perspective, this study delineates a “unipolar superpower-multipolar great power” collaborative framework centered on the United States, with major European nations serving as strategically positioned nodes. The U.S. has established indisputable leadership in global IEI research through its unparalleled dominance across three foundational metrics: publication volume (36.3% global share), citation impact (115,221 citations), and network centrality within collaborative systems. In national productivity rankings, France, the United Kingdom, Italy, and Germany constitute the secondary tier following the United States. This hegemony draws structural reinforcement from sustained contributions of premier institutions including Université Paris Cité, Institut National De La Santé Et De La Recherche Médicale (INSERM), and Assistance Publique Hôpitaux Paris (APHP), alongside seminal scholars such as Jean-Laurent Casanova, Hassan Abolhassani, and Capucine Picard. Their collective intellectual leadership catalyzes institutional and national scholarly prominence through high-impact discovery pipelines. The unipolar architecture, fortified by strategic transnational alliances, ensures accelerated global dissemination, validation, and scaling of IEI breakthroughs, establishing a self-reinforcing innovation cycle. Critical knowledge dissemination hubs include the Journal of Clinical Immunology, Frontiers in Immunology, and Journal of Allergy and Clinical Immunology, which collectively orchestrate the integration of basic research, clinical observations, and therapeutic advancements. Notably, Frontiers in Immunology’s accelerated assimilation of IEI scholarship correlates strongly with its open-access model, streamlined peer-review workflows, and globally diverse authorship. These synergistic mechanisms transform the journal into a dynamic transnational knowledge conduit, exemplifying contemporary imperatives for collaborative, transparent scientific ecosystems. However, this highly centralized research landscape exposes a critical geographical imbalance: the regions where studies are conducted bear little relation to where the clinical burden is likely highest. Whereas North America and Western Europe function as high-density research hubs, vast areas of the Global South remain significant ‘data deserts,’ with populations that are likely underdiagnosed. This disparity constitutes a profound challenge to global health equity, creating an inverse relationship between the need for advanced diagnostics and the availability of relevant research. Prioritizing this imbalance is critical, as outlined in Table S4, to ensure that future innovation addresses the needs of the most underserved populations.

The analysis of research hotspots and keyword burst intensity provides critical insights into the evolutionary trajectory of research focus within the field of IEI. This transition from the historical nomenclature of “Primary Immunodeficiency” to the contemporary IEI framework constitutes more than mere terminological modernization—it signifies a fundamental paradigm shift in both conceptual understanding and scientific priorities. Early investigations centered predominantly on phenotypic characterization and clinical diagnostic criteria for PID. However, transformative advancements in molecular biology, high-throughput sequencing, and genomic technologies have precipitated a comprehensive reconfiguration toward: (1) Elucidating monogenic disease mechanisms; (2) Refining molecular diagnostic modalities; (3) Advancing precision therapeutics and immune reconstitution strategies. This conceptual evolution is intrinsically anchored to the landmark 2019 classification criteria update by the IUIS [31], which systematically codified the expanding clinical spectrum (autoimmune disorders, inflammatory conditions, allergic manifestations, tumor susceptibility) and molecular etiology of IEI. Empirical validation emerges from Thalhammer et al.‘s seminal work [29], demonstrating > 25% diagnostic failure rates when clinicians rely solely on infection-centric symptomatology. Notably, four landmark publications—each achieving citation burst intensities exceeding 80—are directly attributable to IUIS’s iterative nosological refinements (2015–2019) [3, 31–33]. These classification updates have transcended IEI’s traditional association with “recurrent infections,” formally incorporating immune dysregulation phenotypes into diagnostic frameworks. This evidence underscores the imperative for multidimensional diagnostic integration of: (1) Genetic profiling; (2) Immunophenotyping; (3) Clinical biomarker validation. Consequently, the synergistic convergence of technological innovation, nosological standardization, and translational research has inaugurated a transformative era emphasizing molecularly stratified medicine and holistic patient care frameworks in contemporary IEI management.

This paradigmatic shift has not only fundamentally restructured clinical diagnostic pathways for IEI but has also manifested directly in evolving epidemiologic patterns. With the dual advancement of diagnostic technologies and nosological expansion, global IEI patient registries demonstrate substantial growth. Contemporary surveillance data reveal an 86.1% increase in identified cases worldwide (2013–2021), with the United States reporting a 96.3% surge [34]. This trajectory not only contextualizes the U.S.‘s disproportionate research contribution but suggests significant global underdiagnosis—with prevalence estimates approaching 1% in some populations. Among the 15 most prevalent IEIs, CVID predominates globally (13.9%) and nationally in the U.S. (16.6%) [34]. The nosological refinement has similarly catalyzed interdisciplinary convergence, particularly integrating rheumatology, hematology, and oncology. Malignancy susceptibility represents a critical research frontier: among IEI-associated cancers, Primary Antibody Deficiency (PAD: 38.8%, *n* = 1,789), Combined Immunodeficiency with syndromic features (CID: 30.3%, *n* = 1,395), and Bone Marrow Failure (12.7%, *n* = 584) constitute the predominant categories [35]. Molecular profiling identifies ATM protein defects as the most frequent monogenic driver in IEI-related malignancies (20.1%, *n* = 926), while genetically undefined CVID represents the largest non-monogenic cohort (27.9%, *n* = 1,284) [35]—highlighting the imperative for advanced genomic interrogation of complex IEI phenotypes. Therapeutically, immunoglobulin replacement (IVIG) remains foundational, with utilization increasing 110% (2013–2021) and serving approximately 32% of IEI patients [34]. For severe phenotypes, HSCT predominates, with global source distribution: Bone marrow (61%), peripheral blood stem cells (24.4%), and umbilical cord blood (12%) [34]. However, precision therapeutics are revolutionizing management paradigms. Gene therapy emerges as a potentially curative modality, with targeted agents showing promising efficacy. Yet significant translational challenges persist regarding vector safety profiles, delivery optimization, and durable response sustainability—requiring coordinated multidisciplinary innovation.

As a paradigm-shifting exemplar, APDS illustrates the transformative potential of precision medicine in IEI management. This rare monogenic disorder, characterized by overlapping immunodeficiency and lymphoproliferative dysregulation, typically manifests through chronic/recurrent sinopulmonary infections and pathological lymphoid hyperplasia. Despite phenotypic similarities to other IEIs, APDS carries significant mortality (8% case fatality rate, median age of death 18.5 years) [36]. Conventional management remains dependent on lifelong immunoglobulin replacement (IVIG) and broad-spectrum immunosuppression. HSCT demonstrates limited utility - employed in merely 12.8% of patients due to prohibitive risk profiles: 90.9% experience severe adverse events and 36.4% develop graft failure [37, 38].

The therapeutic landscape transformed with the 2022 FDA approval of leniolisib, the first-in-class PI3Kδ inhibitor specifically targeting the disease’s molecular pathogenesis. By selectively inhibiting the hyperactivated PI3Kδ pathway central to APDS immunopathology, it represents a targeted therapeutic watershed [38]. Parallel advances in signaling pathway research continue to accelerate: Recent comprehensive reviews have systematized clinical phenotypes and diagnostic algorithms for JAK-STAT signaling defects [39]; Mounting clinical evidence confirms JAK inhibitors’ efficacy in ameliorating inflammatory manifestations in JAK/STAT-associated IEI, foreshadowing expanded targeted therapeutic arsenals [40]. These breakthroughs underscore the critical imperative for early detection. This direct link between a research hotspot and clinical benefit is even more pronounced in SCID. Our bibliometric analysis identified ‘newborn screening’ as a keyword with significant and sustained citation bursts. This quantitative trend directly mirrors a major clinical success story: a landmark 36-year cohort study demonstrated that universal newborn screening, coupled with timely HSCT, dramatically improves 5-year overall survival in SCID patients from ~ 73% to 87% (P = 0.0005) [41]. Gene therapy now emerges as a revolutionary HSCT-alternative, with validated efficacy across multiple high-mortality IEIs: 1) ADA-SCID, 2) X-linked SCID, 3) Artemis-SCID, 4) Wiskott-Aldrich syndrome, 5) Chronic granulomatous disease (CGD) [9]. This therapeutic continuum—from optimized clinical algorithms to molecularly targeted interventions—not only redefines prognostic possibilities for IEI patients but fundamentally reinvigorates the field’s research trajectory through precision medicine paradigms. This demonstrates a clear pathway where concentrated research investment, identified through bibliometric hotspots, translates directly into life-saving improvements in patient outcomes.

Our analysis also illuminates the varying maturity of therapeutic evidence across different IEIs, as suggested by their research clustering patterns. For SCID, the research is mature, with a dense network of studies on curative options like HSCT and gene therapy. In stark contrast, for CVID—the most prevalent IEI—our analysis shows research remains focused on management (‘immunoglobulin replacement’) and complications, with a noticeable gap in curative strategies, highlighting a major unmet need. APDS serves as an intermediary model: a relatively new but rapidly growing research cluster driven by a deep mechanistic understanding that led to a highly effective precision therapy (leniolisib). This successful ‘bench-to-bedside’ translation for APDS provides a powerful template for tackling other complex IEIs like CVID.

## Key messages for clinicians and policy

The main actionable insights from our study includ: (1) Priority areas for translating research into clinical practice (e.g., expanding access to genetic diagnosis and targeted therapies); (2) Policy recommendations focused on bridging diagnostic and care gaps in under-resourced regions; (3) Identification of IEI subgroups where evidence is most urgent to guide therapy.

## Future research agenda

Our bibliometric analysis highlights notable progress in IEI research but also exposes persistent gaps. To translate scientific advances into global and equitable patient benefit, future efforts should prioritize four interrelated areas.

### Standardized newborn-screening and outcome metrics

The field lacks harmonized indicators for newborn-screening coverage and treatment outcomes. Developing regionally comparable metrics—including quality-adjusted survival, immune reconstitution, and post-HSCT complications—will improve benchmarking and guide clinical decision-making [42].

### Real-world outcomes beyond survival

Long-term real-world outcomes remain understudied. Large prospective cohorts with validated PROMs are needed to assess health-related quality of life and functional impact of different treatments. Systematic tracking of infection and malignancy burden, supported by multicenter or international registries, will enable more comprehensive, patient-centered evaluation [20].

### Long-term safety and economic value of gene therapy

Evidence for gene therapy and gene editing is still limited by small cohorts and short follow-up. Long-term registries and post-marketing surveillance are required to monitor delayed toxicities and assess durability. Comparative effectiveness and cost-effectiveness studies—especially in ADA-SCID, X-SCID, WAS, and CGD—will help determine the true value of these high-cost therapies [13, 43].

### Addressing global south data gaps and building capacity

IEI research is heavily concentrated in the US and Western Europe, leaving significant data gaps elsewhere. Priorities include developing baseline epidemiology, establishing interoperable regional registries, and strengthening diagnostic and research capacity through training and partnerships. Context-appropriate care models—such as simplified diagnostic pathways and tele-immunology—are also needed to support equitable global development [44].

Addressing these priorities will shape the next phase of IEI research and help ensure that scientific progress translates into meaningful and equitable patient benefits worldwide.

## Research materials and methods

We conducted a systematic literature retrieval from the Web of Science Core Collection database using the search query: TS= (“Inborn error of immunity*” OR “Primary immunodeficienc*” OR “Primary immune deficienc*”) with the following parameters: 1) Temporal scope: January 1, 1991 - May 29, 2025; 2) Document types: Articles or Review Articles; 3) Language: English; 4) Exclusions: Retracted publications and book chapters. Bibliometric analysis was performed using: 1) Microsoft Excel 2021: for cataloging publications, cumulative outputs, citation metrics (H-index, G-index, M-index), and total citations; 2) Specialized scientometric tools, including Bibliometrix [45], VOSviewer 1.6.20 [46], CiteSpace 6.2.R6 [47], for visualizing countries, institutions, journals, authors, articles, references, and keywords through trend analysis, cluster identification, co-occurrence networks, and co-citation mapping. We relied on the author identification and disambiguation mechanisms inherent to the Web of Science database.

## Conclusion

This study presents a comprehensive multidimensional visualization of nearly 35 years of IEI research. Through bibliometric analysis of 7,455 articles using complementary visualization tools, we provide an objective assessment of the field’s evolution. Several limitations should be noted: (1) the exclusion of non-Web of Science databases, (2) restriction to English-language publications, and (3) potential underestimation of recently published high-impact studies; (4) the citation trends for recent years are provisional due to citation windows; (5) normalized impact metrics would offer a more robust comparison; (6) the pandemic created a unique confounding surge, making standard growth models less applicable.

Our analysis reveals sustained growth in IEI research, with the field reaching a high-output stability over the past five years, although this observation may be influenced by citation windows and pandemic-related confounding and should not be interpreted as definitive evidence of field maturity or stagnation. Nonetheless, this trajectory suggests that sustaining historical growth rates may require transformative technological advances to drive further advancement. Recent research has prioritized therapeutic innovations – particularly gene therapy, hematopoietic stem cell transplantation (HSCT), and immunoglobulin replacement – while focusing on key diseases: 1) Genetic disorders: CVID, CGD, SCID, leukocyte adhesion deficiency, Wiskott-Aldrich syndrome, ADA deficiency, and APDS; 2) Clinical manifestations: Pneumonia, ataxia-telangiectasia, cancer, asthma, and lymphocytosis.”

## Supplementary Information

Below is the link to the electronic supplementary material.

Supplementary Material 1

Supplementary Material 2

## Data Availability

The original contributions presented in this study are included in the article/supplementary material. Further inquiries can be directed to the corresponding author.

## Abbreviations

ADA: Adenosine deaminase
APDS: Activated PI3Kδ syndrome
APHP: Assistance Publique Hôpitaux Paris
CGD: Chronic granulomatous disease
CVID: Common variable immunodeficiency
GvHD: Graft-versus-host disease
HSC: Hematopoietic stem cell
HSCT: Hematopoietic stem cell transplantation
IEI: Inborn Errors of Immunity
INSERM: Institut National De La Santé Et De La Recherche Médicale
IUIS: International Union of Immunological Societies
JAK: Janus kinase
NGS: Next-generation sequencing
PIDs: Primary Immunodeficiency Diseases
SARS-CoV-2: Severe acute respiratory syndrome coronavirus 2
SCID: Severe combined immunodeficiency
TC: Total citations
TLS: Total Link Strength
VUS: Variants of uncertain significance
XLA: X-linked agammaglobulinemia
