# Supplementary material for: Global research landscape of inborn errors of immunity: a bibliometric analysis (1991–2025)
Source: Orphanet J Rare Dis. 2026 Jan 24;21:64. doi: 10.1186/s13023-025-04191-4 (PMC12911067; doi:10.1186/s13023-025-04191-4)
Supplement: Supplementary file 1 — Supplementary Material 1 [file 13023_2025_4191_MOESM1_ESM.pdf]

Top 50 References with the Strongest Citation Bursts

References	Year	Strength	Begin	End	1991 - 2025
Bonilla FA, 2005, ANN ALLERG ASTHMA IM, V94, PS1, DOI 10.1016/S1081-1206(10)61142-8, DOI	2005	32.31	**2006**	2010	
Notarangelo L, 2006, J ALLERGY CLIN IMMUN, V117, P883, DOI 10.1016/j.jaci.2005.12.1347, DOI	2006	31.13	**2006**	2010	
Salzer U, 2005, NAT GENET, V37, P820, DOI 10.1038/ng1600, DOI	2005	28.78	**2006**	2010	
Holland SM, 2007, NEW ENGL J MED, V357, P1608, DOI 10.1056/NEJMoa073687, DOI	2007	39.38	**2007**	2012	
Minegishi Y, 2006, IMMUNITY, V25, P745, DOI 10.1016/j.immuni.2006.09.009, DOI	2006	28.74	**2007**	2011	
Geha RS, 2007, J ALLERGY CLIN IMMUN, V120, P776, DOI 10.1016/j.jaci.2007.08.053, DOI	2007	59.91	**2008**	2012	
Minegishi Y, 2007, NATURE, V448, P1058, DOI 10.1038/nature06096, DOI	2007	38.94	**2008**	2012	
Milner JD, 2008, NATURE, V452, P773, DOI 10.1038/nature06764, DOI	2008	30.7	**2008**	2013	
Wehr C, 2008, BLOOD, V111, P77, DOI 10.1182/blood-2007-06-091744, DOI	2008	29.1	**2008**	2013	
Chapel H, 2008, BLOOD, V112, P277, DOI 10.1182/blood-2007-11-124545, DOI	2008	31.69	**2009**	2013	
Notarangelo LD, 2009, J ALLERGY CLIN IMMUN, V124, P1161, DOI 10.1016/j.jaci.2009.10.013, DOI	2009	55.82	**2010**	2014	
Zhang Q, 2009, NEW ENGL J MED, V361, P2046, DOI 10.1056/NEJMoa0905506, DOI	2009	30.48	**2010**	2014	
Lucas M, 2010, J ALLERGY CLIN IMMUN, V125, P1354, DOI 10.1016/j.jaci.2010.02.040, DOI	2010	43.88	**2011**	2015	
Orange JS, 2010, CLIN IMMUNOL, V137, P21, DOI 10.1016/j.clim.2010.06.012, DOI	2010	37.15	**2011**	2015	
Gennery AR, 2010, J ALLERGY CLIN IMMUN, V126, P602, DOI 10.1016/j.jaci.2010.06.015, DOI	2010	33.53	**2011**	2015	
Al-Herz W, 2011, FRONT IMMUNOL, V2, P0, DOI 10.3389/fimmu.2011.00054, DOI	2011	68.64	**2012**	2016	
Resnick ES, 2012, BLOOD, V119, P1650, DOI 10.1182/blood-2011-09-377945, DOI	2012	34.21	**2012**	2017	
Liu LY, 2011, J EXP MED, V208, P1635, DOI 10.1084/jem.20110958, DOI	2011	29.68	**2012**	2016	
Lopez-Herrera G, 2012, AM J HUM GENET, V90, P986, DOI 10.1016/j.ajhg.2012.04.015, DOI	2012	30.41	**2013**	2017	
Al-Herz W, 2014, FRONT IMMUNOL, V5, P0, DOI 10.3389/fimmu.2014.00162, DOI	2014	71.62	**2014**	2017	
Lucas CL, 2014, NAT IMMUNOL, V15, P88, DOI 10.1038/ni.2771, DOI	2014	41.74	**2014**	2019	
Angulo I, 2013, SCIENCE, V342, P866, DOI 10.1126/science.1243292, DOI	2013	35.19	**2014**	2018	
Gathmann B, 2014, J ALLERGY CLIN IMMUN, V134, P116, DOI 10.1016/j.jaci.2013.12.1077, DOI	2014	41.82	**2015**	2019	
Schubert D, 2014, NAT MED, V20, P1410, DOI 10.1038/nm.3746, DOI	2014	41.52	**2015**	2019	
Pai SY, 2014, NEW ENGL J MED, V371, P434, DOI 10.1056/NEJMoa1401177, DOI	2014	39.78	**2015**	2019	
Kuehn HS, 2014, SCIENCE, V345, P1623, DOI 10.1126/science.1255904, DOI	2014	37.93	**2015**	2019	
Kwan A, 2014, JAMA-J AM MED ASSOC, V312, P729, DOI 10.1001/jama.2014.9132, DOI	2014	37.49	**2015**	2019	
Picard C, 2015, J CLIN IMMUNOL, V35, P696, DOI 10.1007/s10875-015-0201-1, DOI	2015	85.11	**2016**	2019	
Bonilla FA, 2015, J ALLERGY CLIN IMMUN, V136, P1186, DOI 10.1016/j.jaci.2015.04.049, DOI	2015	46.28	**2016**	2020	
Lo B, 2015, SCIENCE, V349, P436, DOI 10.1126/science.aaa1663, DOI	2015	39.52	**2016**	2020	
Bousfiha A, 2015, J CLIN IMMUNOL, V35, P727, DOI 10.1007/s10875-015-0198-5, DOI	2015	33.19	**2016**	2020	
Bonilla FA, 2016, J ALLER CL IMM-PRACT, V4, P38, DOI 10.1016/j.jaip.2015.07.025, DOI	2016	49.03	**2017**	2021	
Toubiana J, 2016, BLOOD, V127, P3154, DOI 10.1182/blood-2015-11-679902, DOI	2016	36.17	**2017**	2021	

Maffucci P, 2016, FRONT IMMUNOL, V7, P0, DOI 10.3389/fimmu.2016.00220, DOI	2016	28.84	**2017**	2021
Picard C, 2018, J CLIN IMMUNOL, V38, P96, DOI 10.1007/s10875-017-0464-9, DOI	2018	84.76	**2018**	2021
Bousfiha A, 2018, J CLIN IMMUNOL, V38, P129, DOI 10.1007/s10875-017-0465-8, DOI	2018	53.64	**2018**	2021
Coulter TI, 2017, J ALLERGY CLIN IMMUN, V139, P597, DOI 10.1016/j.jaci.2016.06.021, DOI	2017	33.82	**2018**	2022
Stray-Pedersen A, 2017, J ALLERGY CLIN IMMUN, V139, P232, DOI 10.1016/j.jaci.2016.05.042, DOI	2017	31.62	**2018**	2022
Schwab C, 2018, J ALLERGY CLIN IMMUN, V142, P1932, DOI 10.1016/j.jaci.2018.02.055, DOI	2018	40.27	**2019**	2023
Fischer A, 2017, J ALLERGY CLIN IMMUN, V140, P1388, DOI 10.1016/j.jaci.2016.12.978, DOI	2017	28.7	**2019**	2022
Tangye SG, 2020, J CLIN IMMUNOL, V40, P24, DOI 10.1007/s10875-019-00737-x, DOI	2020	149.72	**2020**	2023
Seidel MG, 2019, J ALLER CL IMM-PRACT, V7, P1763, DOI 10.1016/j.jaip.2019.02.004, DOI	2019	62.06	**2020**	2025
Bousfiha A, 2020, J CLIN IMMUNOL, V40, P66, DOI 10.1007/s10875-020-00758-x, DOI	2020	84.84	**2021**	2023
Tangye SG, 2021, J CLIN IMMUNOL, V41, P666, DOI 10.1007/s10875-021-00980-1, DOI	2021	43.24	**2021**	2023
Meyts I, 2021, J ALLERGY CLIN IMMUN, V147, P520, DOI 10.1016/j.jaci.2020.09.010, DOI	2021	39.8	**2021**	2025
Notarangelo LD, 2020, SCI IMMUNOL, V5, P0, DOI 10.1126/sciimmunol.abb1662, DOI	2020	36.33	**2021**	2025
Zhang Q, 2020, SCIENCE, V370, P0, DOI 10.1126/science.abd4570, DOI	2020	35.98	**2021**	2025
Bastard P, 2020, SCIENCE, V370, P423, DOI 10.1126/science.abd4585, DOI	2020	34.24	**2021**	2025
Abolhassani H, 2020, BLOOD, V135, P656, DOI 10.1182/blood.2019000929, DOI	2020	30.08	**2021**	2025
Thalhammer J, 2021, J ALLERGY CLIN IMMUN, V148, P1332, DOI 10.1016/j.jaci.2021.04.015, DOI	2021	31.44	**2022**	2025
