# Supplementary material for: Global research landscape of inborn errors of immunity: a bibliometric analysis (1991–2025)
Source: Orphanet J Rare Dis. 2026 Jan 24;21:64. doi: 10.1186/s13023-025-04191-4 (PMC12911067; doi:10.1186/s13023-025-04191-4)
Supplement: Supplementary file 2 — Supplementary Material 2 [file 13023_2025_4191_MOESM2_ESM.docx]

**Supplementary material 1**

Figure S1. Publication trends in the United States (1991-2024) 2

Figure S2. The total link strength of the national collaboration network analysis 3

Figure S3. Country production over time 4

Figure S4. Corresponding author’s countries 5

Figure S5. Country collaboration map 6

Figure S6. The top 20 publications of the affiliations (1991-2025) 7

Figure S7. The 20 most relevant affiliations 8

Figure S8. The 20 most relevant sources 9

Figure S9. The total link strength of the journals network analysis 10

Figure S10. Sources’ production over time 11

Figure S11. The 20 most local cited sources 12

Figure S12. Sources’ local impact by H-index 13

Figure S13. The 20 most relevant authors 14

Figure S14. The 20 most local cited authors 15

Figure S15. Authors’ production over time 16

Table S1. The top 20 authors’ impact by H-index 17

Figure S16. The total link strength of the authors network analysis 18

Figure S17. The total link strength of the co-cited authors network analysis 19

Table S2. The top 20 cited articles 20

Table S3. The cited reference at least 100 times 21-25

Figure S18. Co-citation network analysis of references 26

Figure S19. The 100 most frequent keywords 17

Figure S20. The trend of keywords for topics 28

Figure S21. Top 50 keywords with the strongest citation bursts 29

Table S4. Regional comparison of IEI research, challenges, and recommended actions 30

**Figure S1. Publication trends in the United States (1991-2024)**

**Figure S2.** **The total link strength of the national collaboration network analysis**

**
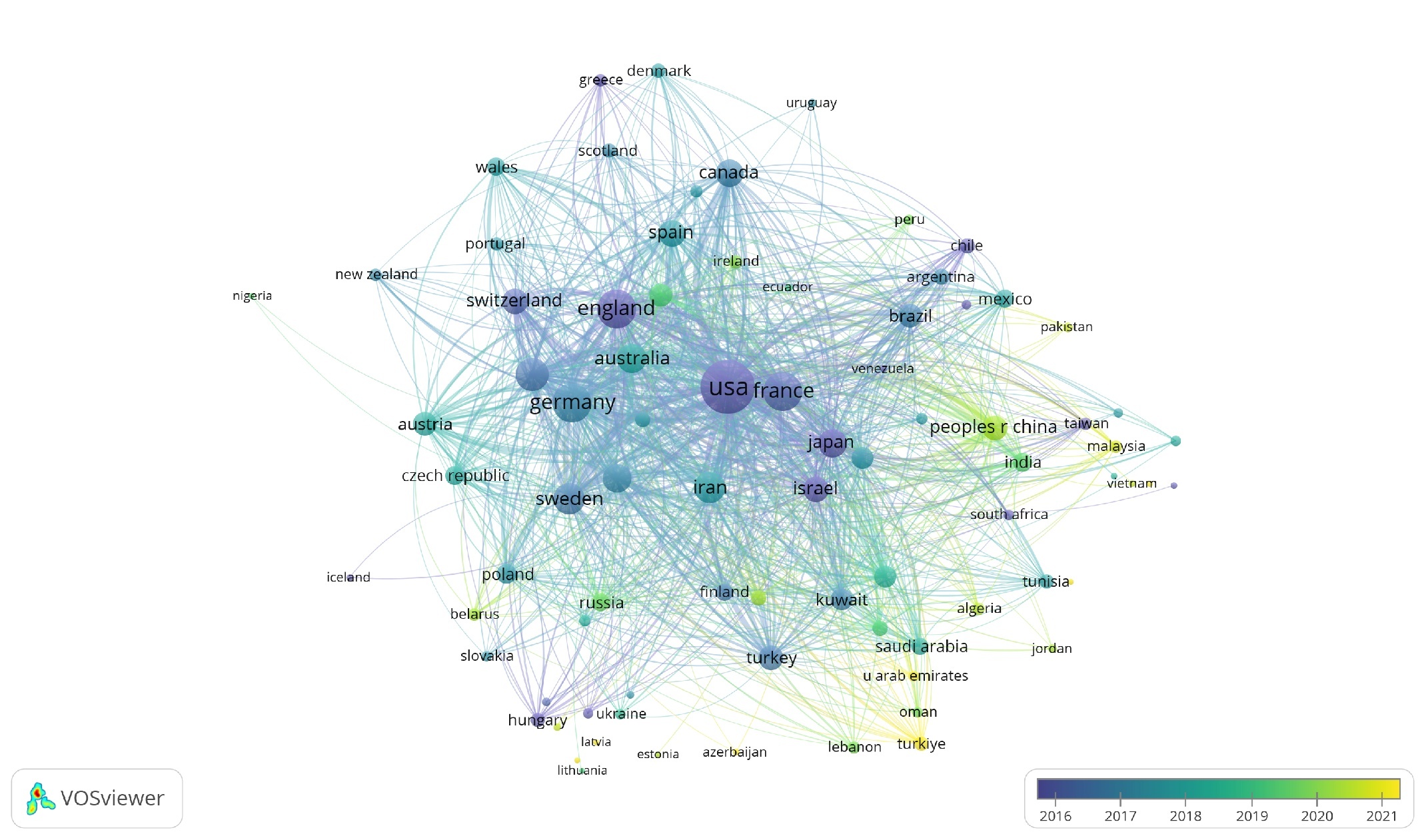
**

**Figure S3. Country production over time**


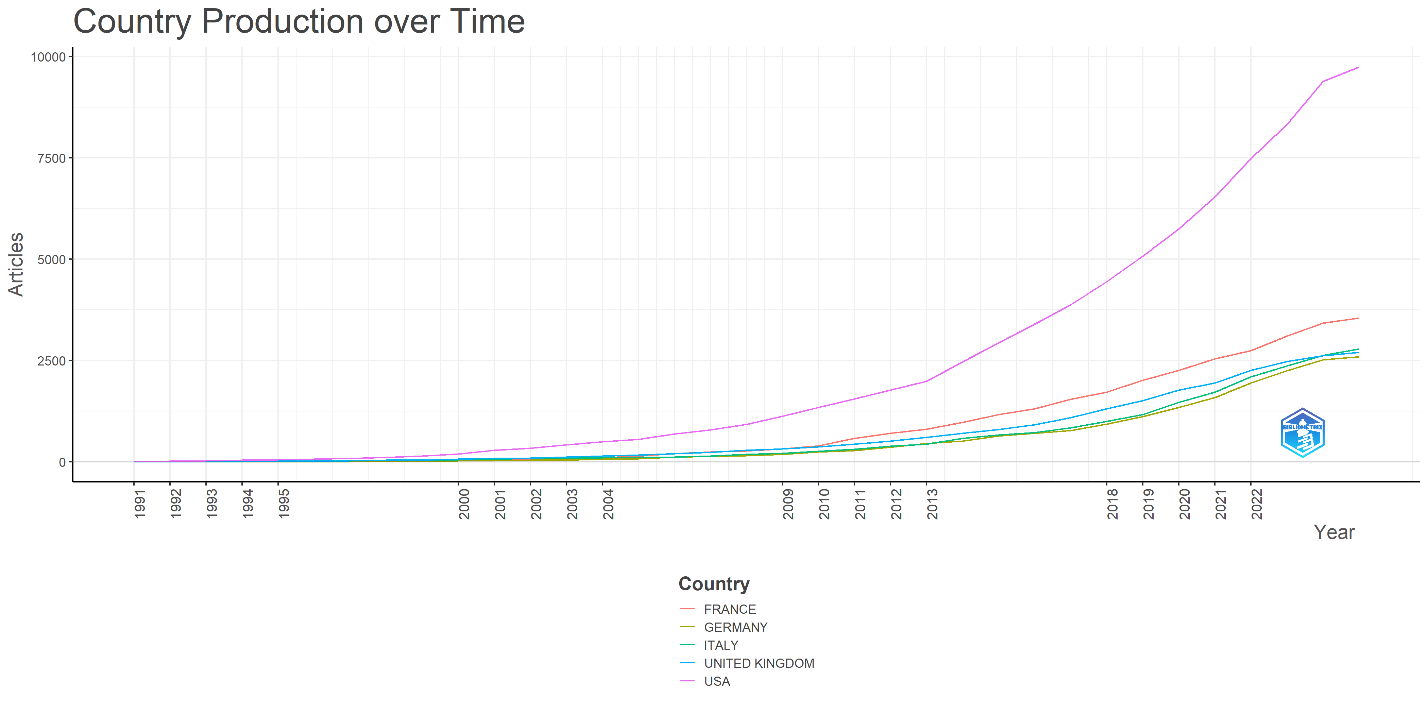


**Figure S4.** **Corresponding author’s countries**

**
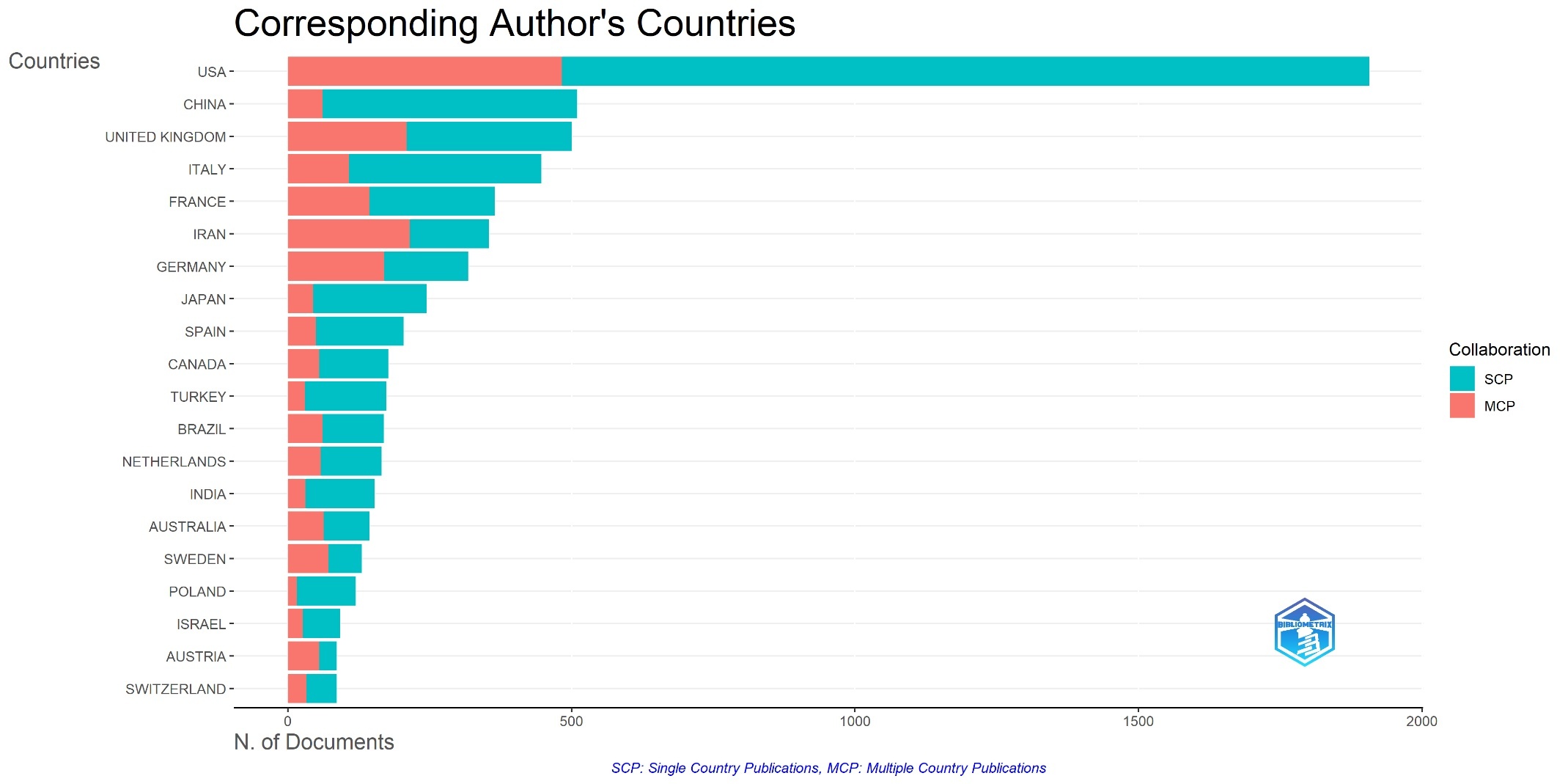
**

**Figure S5. Country collaboration map**

**
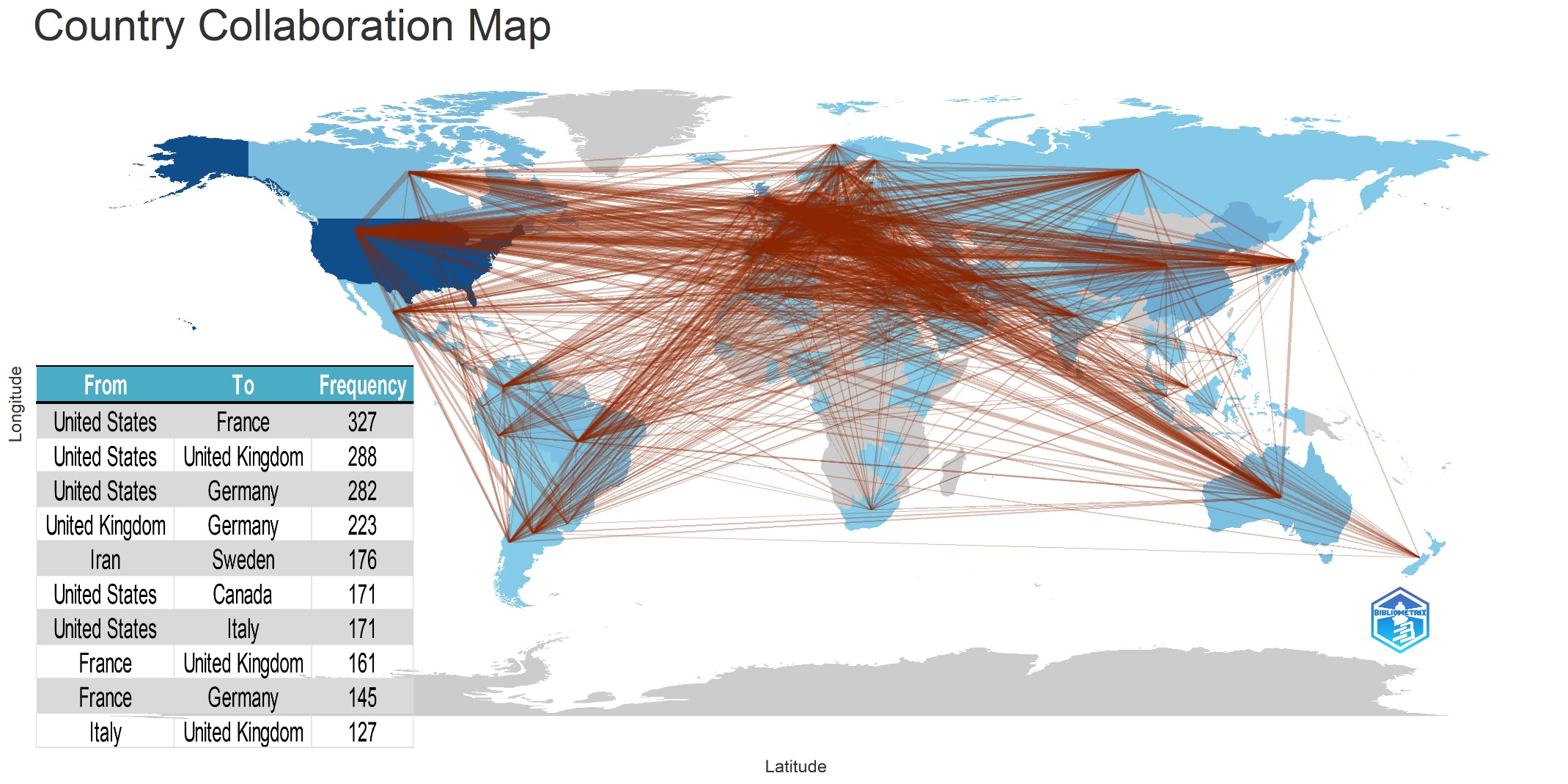
**

**Figure S6. The top 20 publications of the affiliations (1991-2025)**

**Figure S7. The 20 most relevant affiliations**


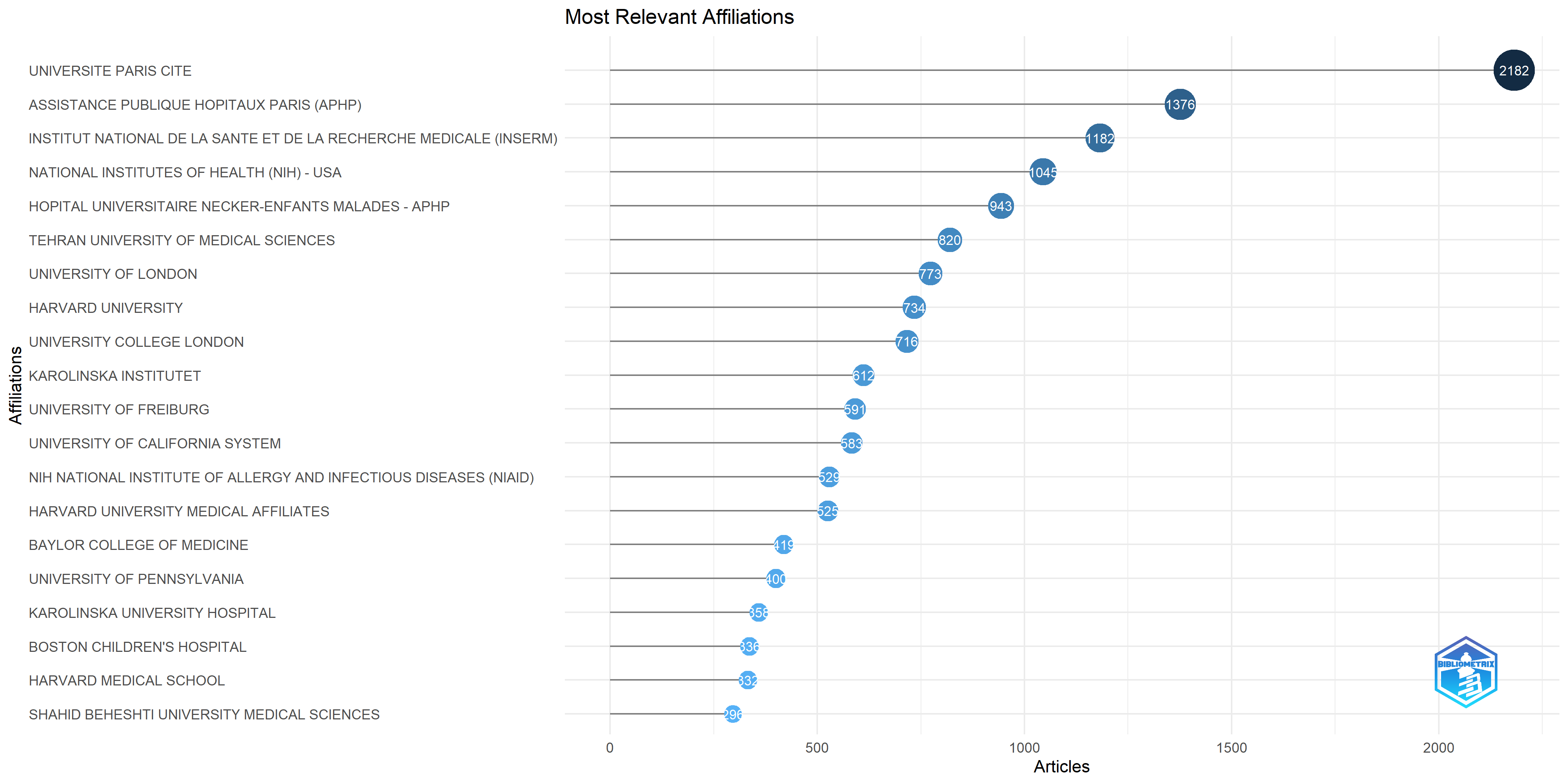


**Figure S8. The 20 most relevant sources**


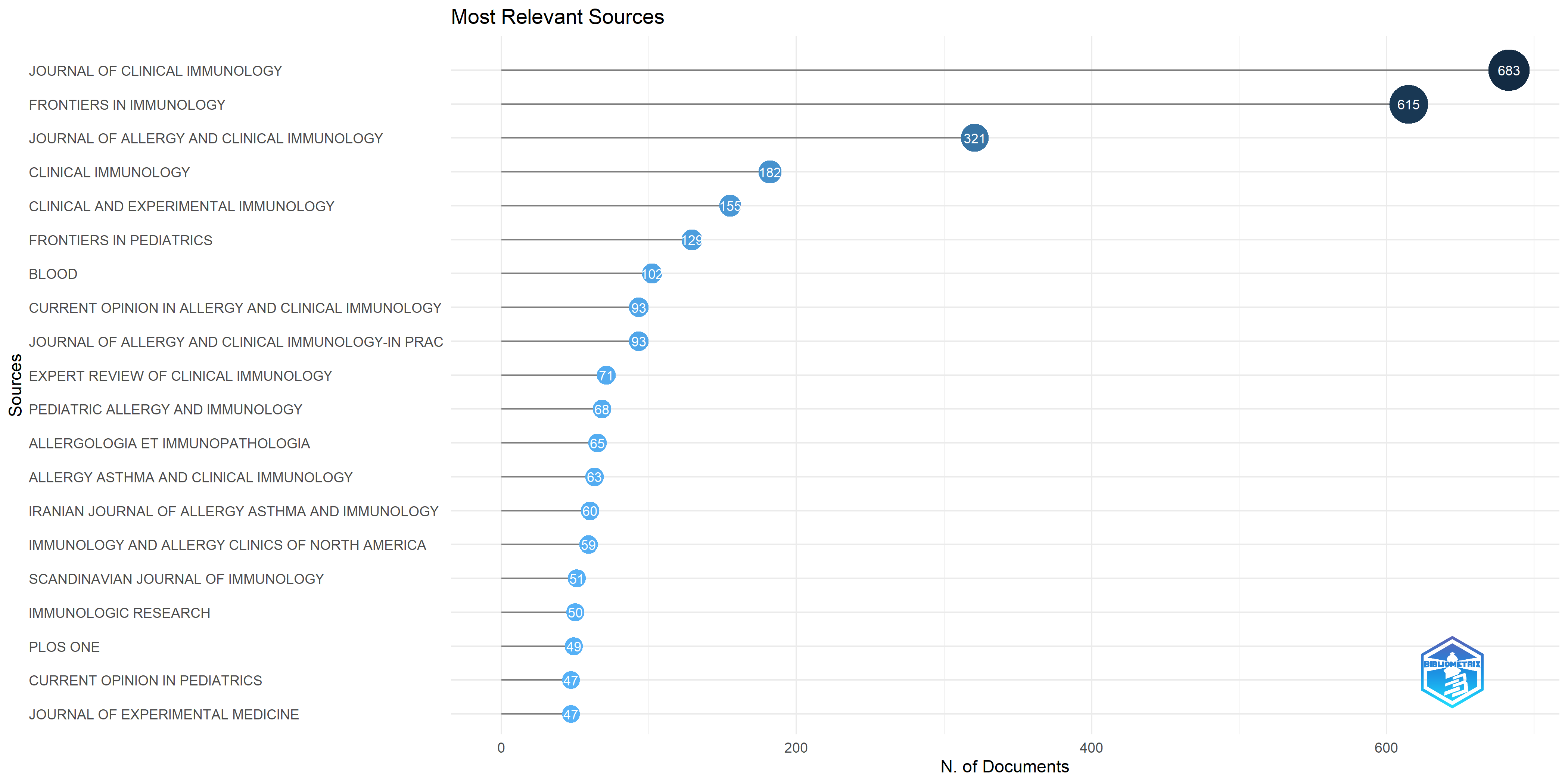


**Figure S9. The total link strength of the journals network analysis**

**
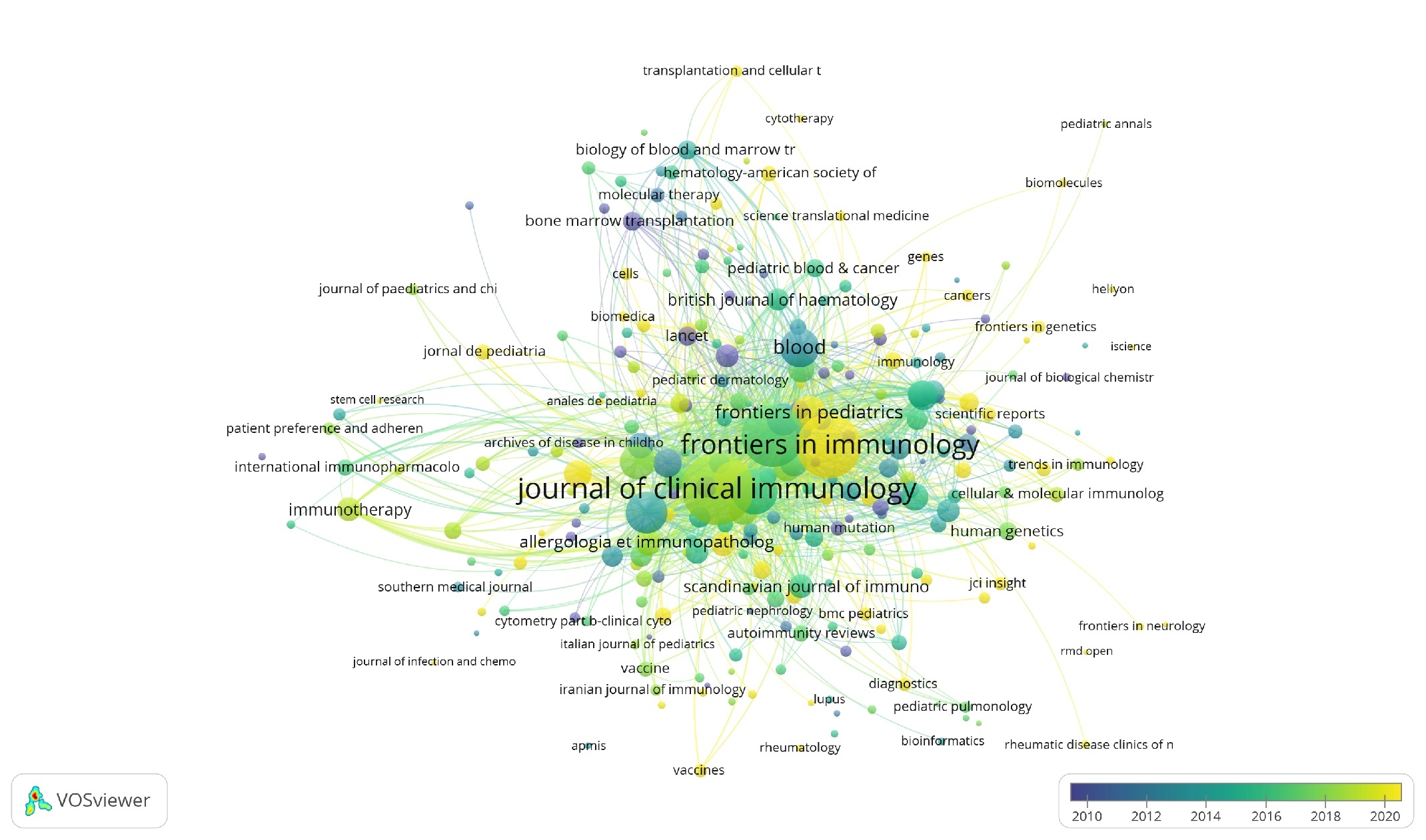
**

**Figure S10. Sources’ production over time**


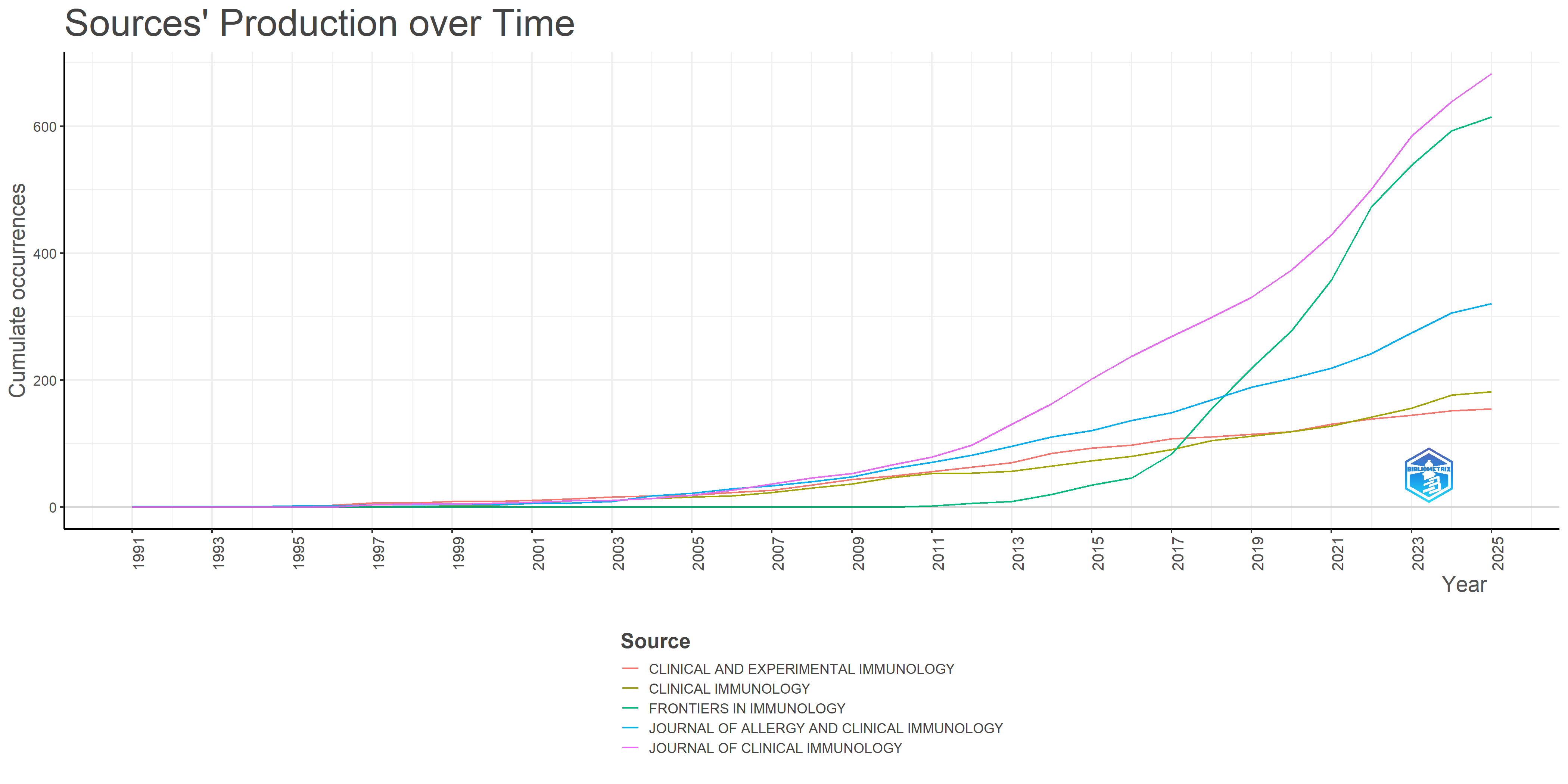


**Figure S11. The 20 most local cited sources**


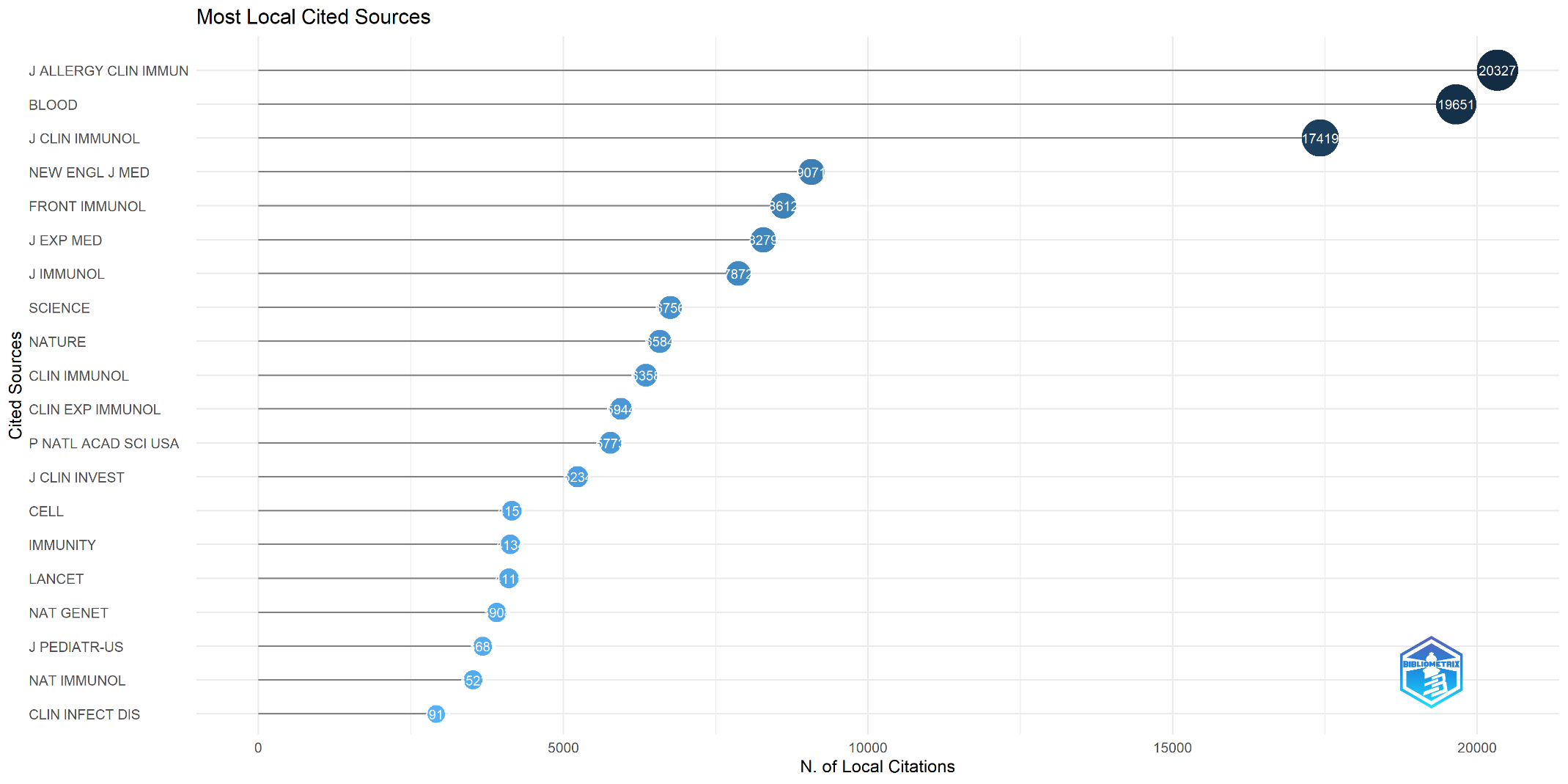


**Figure S12. Sources’ local impact by H-index**


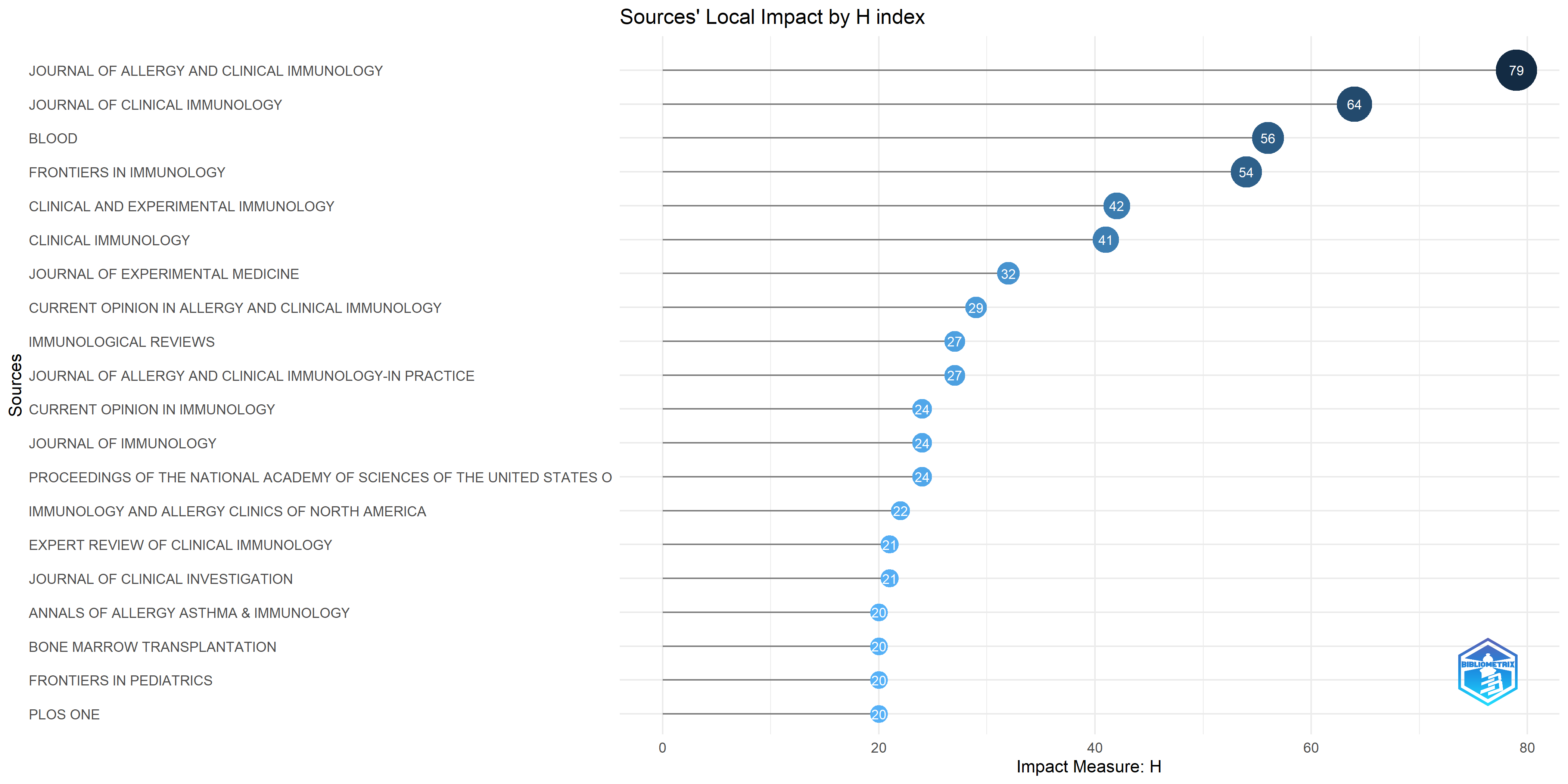


**Figure S13. The 20 most relevant authors**


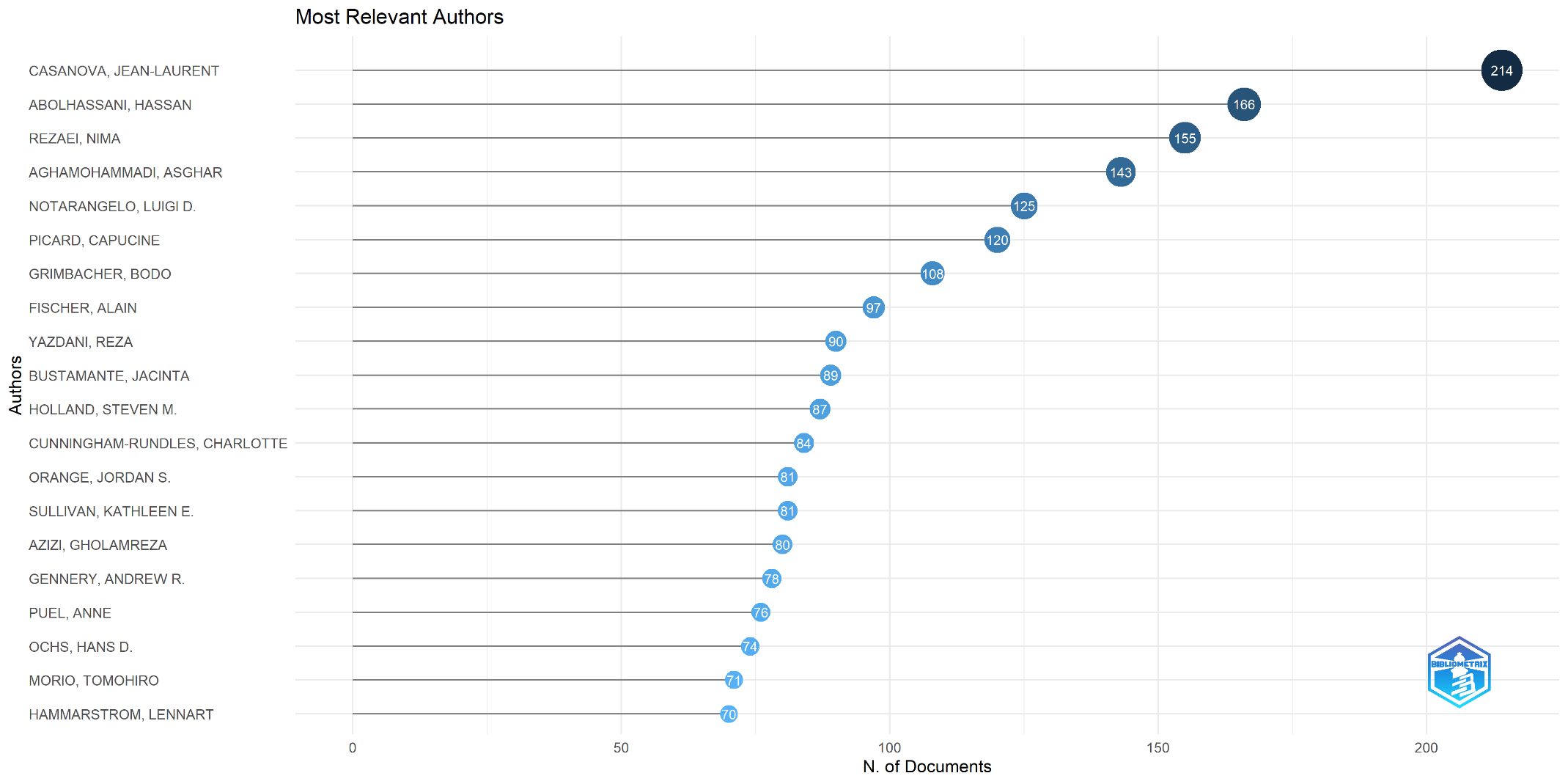


**Figure S14. The 20 most local cited authors**


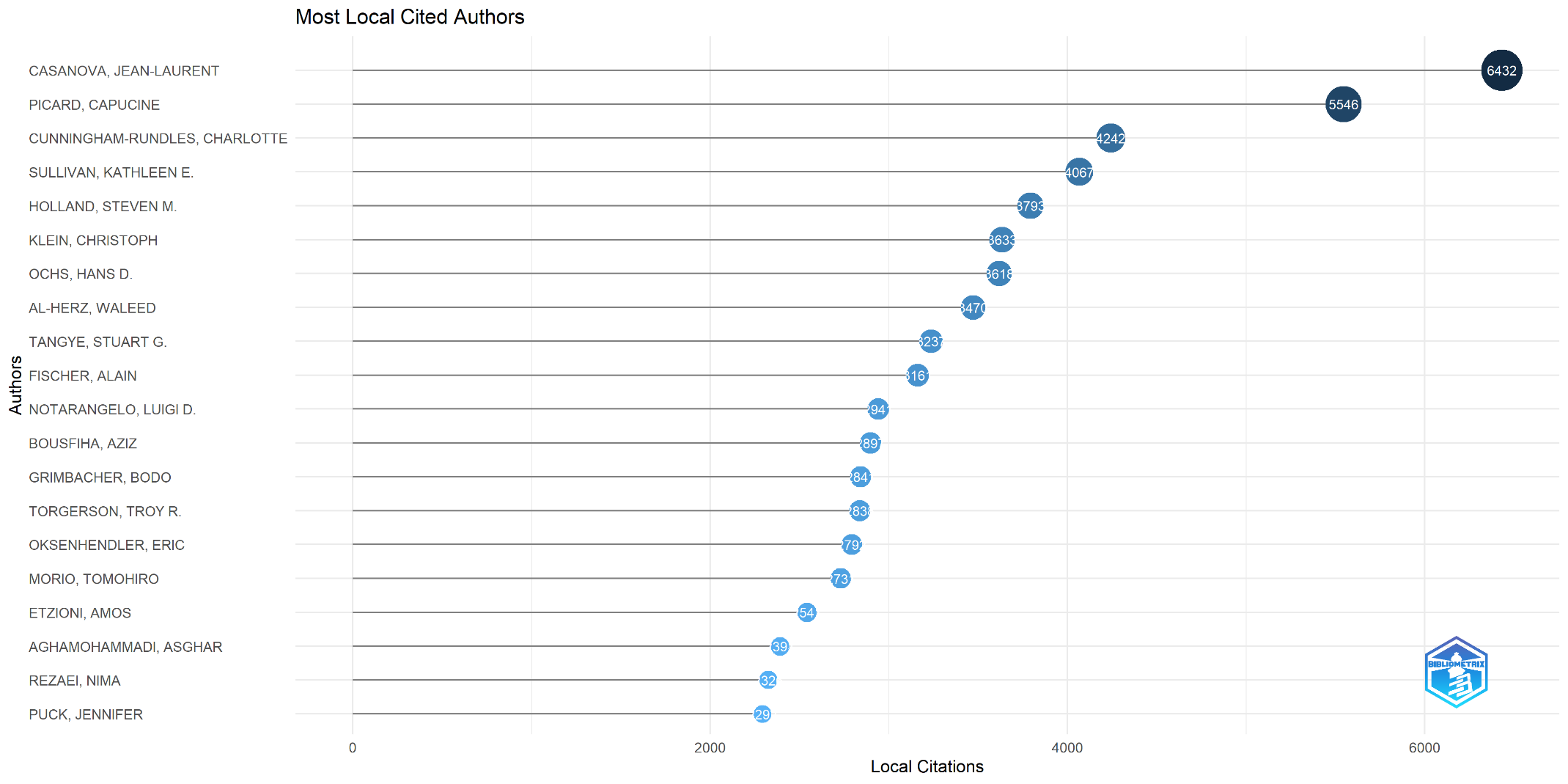


**Figure S15. Authors’ production over time**


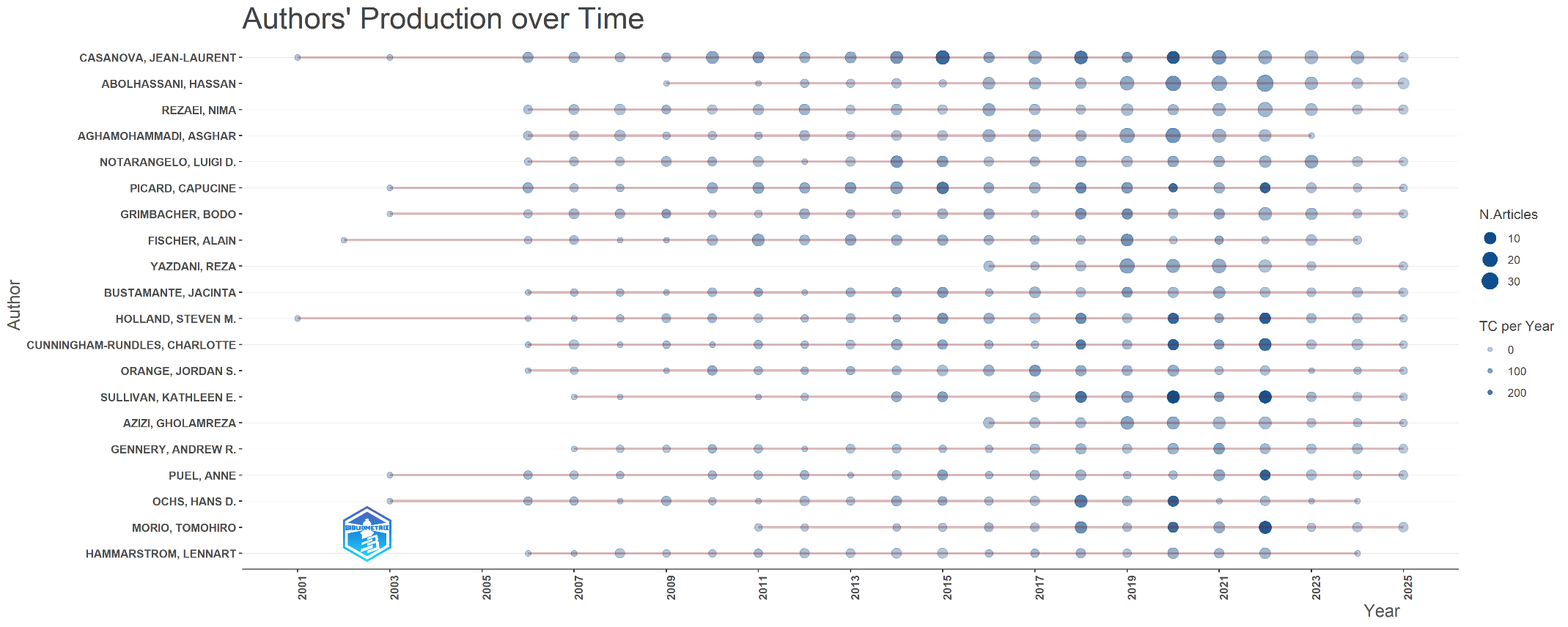


**Table S1. The top 20 authors’ impact by H-index**

| **Author** | **H-Index** | **G-Index** | **M-Index** | **Total Citation** | **Publications** | **Year of publication start** |
| --- | --- | --- | --- | --- | --- | --- |
| CASANOVA JEAN-LAURENT | 72 | 132 | 2.88 | 18242 | 214 | 2001 |
| PICARD CAPUCINE | 59 | 119 | 2.57 | 14280 | 120 | 2003 |
| ABEL LAURENT | 45 | 66 | 2.25 | 7746 | 66 | 2006 |
| HOLLAND STEVEN M. | 45 | 87 | 1.80 | 8718 | 87 | 2001 |
| FISCHER ALAIN | 44 | 85 | 1.83 | 7350 | 97 | 2002 |
| GRIMBACHER BODO | 44 | 88 | 1.91 | 7838 | 108 | 2003 |
| NOTARANGELO LUIGI D. | 43 | 82 | 2.15 | 7119 | 125 | 2006 |
| PUEL ANNE | 40 | 76 | 1.74 | 6479 | 76 | 2003 |
| AGHAMOHAMMADI ASGHAR | 39 | 61 | 1.95 | 4558 | 143 | 2006 |
| CUNNINGHAM-RUNDLES CHARLOTTE | 39 | 84 | 1.95 | 8513 | 84 | 2006 |
| ORANGE JORDAN S. | 39 | 75 | 1.95 | 5674 | 81 | 2006 |
| SULLIVAN KATHLEEN E. | 39 | 81 | 2.05 | 7922 | 81 | 2007 |
| OCHS HANS D. | 38 | 74 | 1.65 | 7253 | 74 | 2003 |
| REZAEI NIMA | 38 | 71 | 1.90 | 5613 | 155 | 2006 |
| PUCK JENNIFER M. | 37 | 53 | 1.68 | 5121 | 53 | 2004 |
| TANGYE STUART G. | 37 | 67 | 2.06 | 7570 | 67 | 2008 |
| BUSTAMANTE JACINTA | 35 | 77 | 1.75 | 6085 | 89 | 2006 |
| ABOLHASSANI HASSAN | 34 | 56 | 2.00 | 3965 | 166 | 2009 |
| KLEIN CHRISTOPH | 34 | 62 | 1.70 | 7902 | 62 | 2006 |
| AL-HERZ WALEED | 32 | 54 | 1.78 | 6530 | 54 | 2008 |

**Figure S16. The total link strength of the authors network analysis**

**
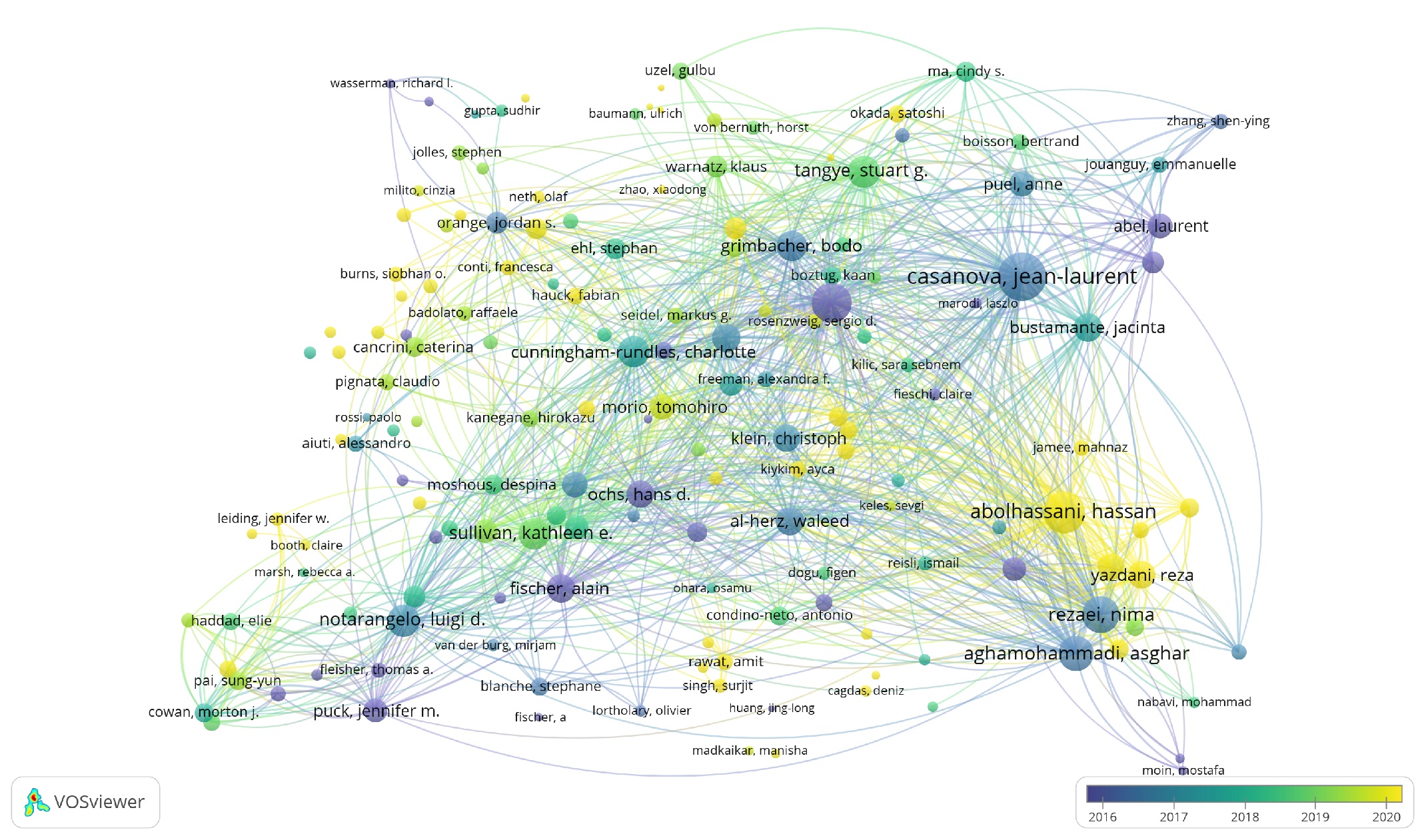
**

**Figure S17. The total link strength of the co-cited authors network analysis**

**
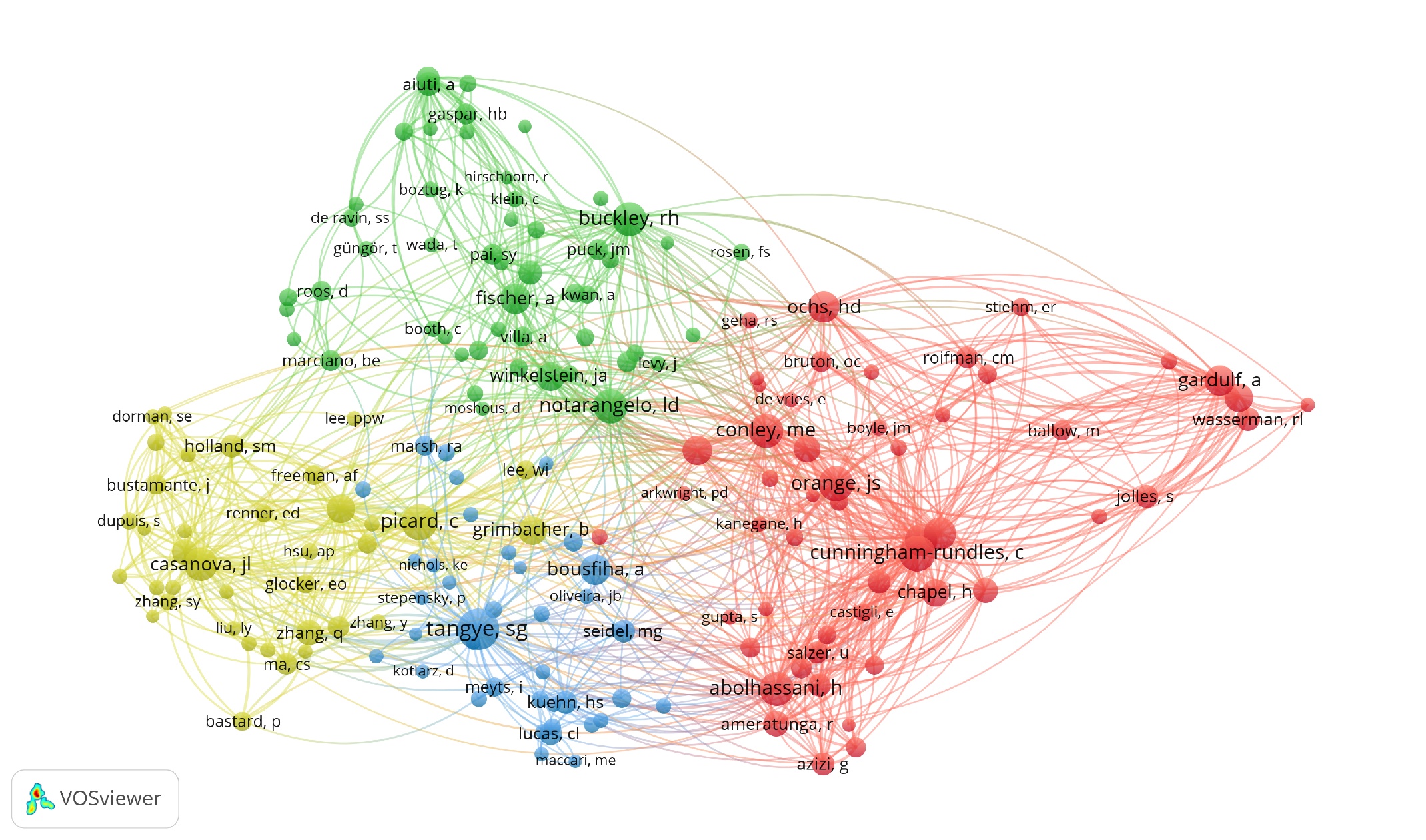
**

**Table S2. The top 20 cited articles**

| **Articles** | **DOI** | **Total Citations (TC)** | **TC per Year** | **Normalized TC** |
| --- | --- | --- | --- | --- |
| MOGENSEN TH, 2009, CLIN MICROBIOL REV | 10.1128/CMR.00046-08 | 2205 | 129.71 | 29.98 |
| VIVIER E, 2011, SCIENCE | 10.1126/science.1198687 | 2041 | 136.07 | 35.08 |
| OKADA Y, 2014, NATURE | 10.1038/nature12873 | 1718 | 143.17 | 38.41 |
| CUNNINGHAM-RUNDLES C, 1999, CLIN IMMUNOL | 10.1006/clim.1999.4725 | 1172 | 43.41 | 9.59 |
| WINKELSTEIN JA, 2000, MEDICINE | 10.1097/00005792-200005000-00003 | 1161 | 44.65 | 20.80 |
| DE LANGE KM, 2017, NAT GENET | 10.1038/ng.3760 | 921 | 102.33 | 26.38 |
| OTT MG, 2006, NAT MED | 10.1038/nm1393 | 918 | 45.90 | 12.98 |
| GHORESCHI K, 2009, IMMUNOL REV | 10.1111/j.1600-065X.2008.00754.x | 916 | 53.88 | 12.45 |
| CONLEY ME, 1999, CLIN IMMUNOL | 10.1006/clim.1999.4799 | 864 | 32.00 | 7.07 |
| TANGYE SG, 2020, J CLIN IMMUNOL | 10.1007/s10875-019-00737-x | 856 | 142.67 | 36.21 |
| MINEGISHI Y, 2007, NATURE | 10.1038/nature06096 | 804 | 42.32 | 15.15 |
| STEIMLE V, 1993, CELL | 10.1016/0092-8674(93)90685-J | 798 | 24.18 | 10.54 |
| PALMEIRA P, 2012, CLIN DEV IMMUNOL | 10.1155/2012/985646 | 692 | 49.43 | 14.31 |
| TANGYE SG, 2022, J CLIN IMMUNOL | 10.1007/s10875-022-01289-3 | 668 | 167.00 | 59.68 |
| DISANTO JP, 1993, NATURE | 10.1038/361541a0 | 656 | 19.88 | 8.67 |
| SHEARER WT, 2003, J ALLERGY CLIN IMMUN | 10.1016/j.jaci.2003.07.003 | 651 | 28.30 | 9.66 |
| GLOCKER EO, 2009, NEW ENGL J MED | 10.1056/NEJMoa0810719 | 636 | 37.41 | 8.65 |
| MILONE MC, 2018, LEUKEMIA | 10.1038/s41375-018-0106-0 | 607 | 75.88 | 16.43 |
| CASROUGE A, 2006, SCIENCE | 10.1126/science.1128346 | 600 | 30.00 | 8.48 |
| PICARD C, 2018, J CLIN IMMUNOL | 10.1007/s10875-017-0464-9 | 580 | 72.50 | 15.70 |

**Table S3. The cited reference at least 100 times**

| **Cited Reference** | **Total citations** | **Total link strengths** |
| --- | --- | --- |
| cunningham-rundles c, 1999, clin immunol, v92, p34, doi 10.1006/clim.1999.4725 | 387 | 2446 |
| tangye sg, 2020, j clin immunol, v40, p24, doi 10.1007/s10875-019-00737-x | 512 | 2128 |
| resnick es, 2012, blood, v119, p1650, doi 10.1182/blood-2011-09-377945 | 259 | 2052 |
| chapel h, 2008, blood, v112, p277, doi 10.1182/blood-2007-11-124545 | 254 | 2042 |
| minegishi y, 2007, nature, v448, p1058, doi 10.1038/nature06096 | 245 | 1829 |
| wehr c, 2008, blood, v111, p77, doi 10.1182/blood-2007-06-091744 | 240 | 1827 |
| holland sm, 2007, new engl j med, v357, p1608, doi 10.1056/nejmoa073687 | 241 | 1757 |
| bonilla fa, 2016, j aller cl imm-pract, v4, p38, doi 10.1016/j.jaip.2015.07.025 | 243 | 1712 |
| schubert d, 2014, nat med, v20, p1410, doi 10.1038/nm.3746 | 195 | 1684 |
| bruton oc, 1952, pediatrics, v9, p722 | 303 | 1665 |
| lucas cl, 2014, nat immunol, v15, p88, doi 10.1038/ni.2771 | 215 | 1652 |
| kuehn hs, 2014, science, v345, p1623, doi 10.1126/science.1255904 | 181 | 1638 |
| conley me, 1999, clin immunol, v93, p190, doi 10.1006/clim.1999.4799 | 316 | 1589 |
| lopez-herrera g, 2012, am j hum genet, v90, p986, doi 10.1016/j.ajhg.2012.04.015 | 179 | 1582 |
| picard c, 2018, j clin immunol, v38, p96, doi 10.1007/s10875-017-0464-9 | 324 | 1571 |
| lo b, 2015, science, v349, p436, doi 10.1126/science.aaa1663 | 182 | 1540 |
| gathmann b, 2014, j allergy clin immun, v134, p116, doi 10.1016/j.jaci.2013.12.1077 | 208 | 1497 |
| angulo i, 2013, science, v342, p866, doi 10.1126/science.1243292 | 194 | 1487 |
| liu ly, 2011, j exp med, v208, p1635, doi 10.1084/jem.20110958 | 178 | 1430 |
| grimbacher b, 2003, nat immunol, v4, p261, doi 10.1038/ni902 | 159 | 1389 |
| quinti i, 2007, j clin immunol, v27, p308, doi 10.1007/s10875-007-9075-1 | 170 | 1362 |
| grimbacher b, 1999, new engl j med, v340, p692, doi 10.1056/nejm199903043400904 | 205 | 1340 |
| milner jd, 2015, blood, v125, p591, doi 10.1182/blood-2014-09-602763 | 145 | 1332 |
| schwab c, 2018, j allergy clin immun, v142, p1932, doi 10.1016/j.jaci.2018.02.055 | 174 | 1330 |
| warnatz k, 2002, blood, v99, p1544, doi 10.1182/blood.v99.5.1544 | 157 | 1277 |
| tangye sg, 2022, j clin immunol, v42, p1473, doi 10.1007/s10875-022-01289-3 | 384 | 1254 |
| salzer u, 2005, nat genet, v37, p820, doi 10.1038/ng1600 | 138 | 1244 |
| zhang q, 2009, new engl j med, v361, p2046, doi 10.1056/nejmoa0905506 | 191 | 1241 |
| winkelstein ja, 2000, medicine, v79, p155, doi 10.1097/00005792-200005000-00003 | 313 | 1228 |
| lucas m, 2010, j allergy clin immun, v125, p1354, doi 10.1016/j.jaci.2010.02.040 | 177 | 1227 |
| coulter ti, 2017, j allergy clin immun, v139, p597, doi 10.1016/j.jaci.2016.06.021 | 167 | 1227 |
| toubiana j, 2016, blood, v127, p3154, doi 10.1182/blood-2015-11-679902 | 159 | 1205 |
| milner jd, 2008, nature, v452, p773, doi 10.1038/nature06764 | 150 | 1205 |
| castigli e, 2005, nat genet, v37, p829, doi 10.1038/ng1601 | 128 | 1184 |
| pai sy, 2014, new engl j med, v371, p434, doi 10.1056/nejmoa1401177 | 227 | 1142 |
| van zelm mc, 2006, new engl j med, v354, p1901, doi 10.1056/nejmoa051568 | 128 | 1136 |
| van de veerdonk fl, 2011, new engl j med, v365, p54, doi 10.1056/nejmoa1100102 | 132 | 1122 |
| bonilla fa, 2015, j allergy clin immun, v136, p1186, doi 10.1016/j.jaci.2015.04.049 | 226 | 1121 |
| oksenhendler e, 2008, clin infect dis, v46, p1547, doi 10.1086/587669 | 141 | 1101 |
| bousfiha a, 2020, j clin immunol, v40, p66, doi 10.1007/s10875-020-00758-x | 269 | 1097 |
| orange js, 2010, clin immunol, v137, p21, doi 10.1016/j.clim.2010.06.012 | 187 | 1097 |
| boyle jm, 2007, j clin immunol, v27, p497, doi 10.1007/s10875-007-9103-1 | 211 | 1051 |
| engelhardt kr, 2009, j allergy clin immun, v124, p1289, doi 10.1016/j.jaci.2009.10.038 | 145 | 1051 |
| winkelstein ja, 2003, medicine, v82, p373, doi 10.1097/01.md.0000100046.06009.b0 | 159 | 1026 |
| seidel mg, 2019, j aller cl imm-pract, v7, p1763, doi 10.1016/j.jaip.2019.02.004 | 235 | 1008 |
| minegishi y, 2006, immunity, v25, p745, doi 10.1016/j.immuni.2006.09.009 | 136 | 1007 |
| winkelstein ja, 2006, medicine, v85, p193, doi 10.1097/01.md.0000229482.27398.ad | 148 | 1003 |
| vetrie d, 1993, nature, v361, p226, doi 10.1038/361226a0 | 167 | 999 |
| picard c, 2015, j clin immunol, v35, p696, doi 10.1007/s10875-015-0201-1 | 247 | 989 |
| bonilla fa, 2005, ann allerg asthma im, v94, ps1, doi 10.1016/s1081-1206(10)61142-8 | 182 | 970 |
| maffucci p, 2016, front immunol, v7, doi 10.3389/fimmu.2016.00220 | 125 | 968 |
| levy j, 1997, j pediatr-us, v131, p47, doi 10.1016/s0022-3476(97)70123-9 | 175 | 934 |
| rao vk, 2017, blood, v130, p2307, doi 10.1182/blood-2017-08-801191 | 110 | 931 |
| chapel h, 2009, brit j haematol, v145, p709, doi 10.1111/j.1365-2141.2009.07669.x | 107 | 920 |
| elkaim e, 2016, j allergy clin immun, v138, p210, doi 10.1016/j.jaci.2016.03.022 | 114 | 916 |
| kwan a, 2014, jama-j am med assoc, v312, p729, doi 10.1001/jama.2014.9132 | 186 | 902 |
| gámez-díaz l, 2016, j allergy clin immun, v137, p223, doi 10.1016/j.jaci.2015.09.025 | 106 | 895 |
| busse pj, 2002, j allergy clin immun, v109, p1001, doi 10.1067/mai.2002.124999 | 120 | 887 |
| davis sd, 1966, lancet, v1, p1013 | 122 | 885 |
| ma cs, 2008, j exp med, v205, p1551, doi 10.1084/jem.20080218 | 109 | 869 |
| sullivan ke, 1994, j pediatr-us, v125, p876, doi 10.1016/s0022-3476(05)82002-5 | 166 | 863 |
| bousfiha a, 2018, j clin immunol, v38, p129, doi 10.1007/s10875-017-0465-8 | 199 | 862 |
| al-herz w, 2014, front immunol, v5, doi 10.3389/fimmu.2014.00162 | 170 | 858 |
| tsukada s, 1993, cell, v72, p279, doi 10.1016/0092-8674(93)90667-f | 136 | 858 |
| gennery ar, 2010, j allergy clin immun, v126, p602, doi 10.1016/j.jaci.2010.06.015 | 166 | 854 |
| hermaszewski ra, 1993, q j med, v86, p31 | 127 | 845 |
| bogaert dja, 2016, j med genet, v53, p575, doi 10.1136/jmedgenet-2015-103690 | 109 | 842 |
| lucas cl, 2014, j exp med, v211, p2537, doi 10.1084/jem.20141759 | 108 | 834 |
| fliegauf m, 2015, am j hum genet, v97, p389, doi 10.1016/j.ajhg.2015.07.008 | 103 | 823 |
| fischer a, 2017, j allergy clin immun, v140, p1388, doi 10.1016/j.jaci.2016.12.978 | 137 | 812 |
| orange js, 2006, j allergy clin immun, v117, ps525, doi 10.1016/j.jaci.2006.01.015 | 150 | 786 |
| notarangelo ld, 2009, j allergy clin immun, v124, p1161, doi 10.1016/j.jaci.2009.10.013 | 142 | 779 |
| puel a, 2011, science, v332, p65, doi 10.1126/science.1200439 | 104 | 776 |
| grimbacher b, 1999, am j hum genet, v65, p735, doi 10.1086/302547 | 134 | 764 |
| plebani a, 2002, clin immunol, v104, p221, doi 10.1006/clim.2002.5241 | 113 | 761 |
| stray-pedersen a, 2017, j allergy clin immun, v139, p232, doi 10.1016/j.jaci.2016.05.042 | 136 | 755 |
| puel a, 2010, j exp med, v207, p291, doi 10.1084/jem.20091983 | 112 | 753 |
| chandesris mo, 2012, medicine, v91, pe1, doi 10.1097/md.0b013e31825f95b9 | 111 | 747 |
| bennett cl, 2001, nat genet, v27, p20, doi 10.1038/83713 | 108 | 741 |
| geha rs, 2007, j allergy clin immun, v120, p776, doi 10.1016/j.jaci.2007.08.053 | 162 | 714 |
| quinti i, 2011, j clin immunol, v31, p315, doi 10.1007/s10875-011-9511-0 | 108 | 712 |
| bousfiha aa, 2013, j clin immunol, v33, p1, doi 10.1007/s10875-012-9751-7 | 147 | 708 |
| renner ed, 2004, j pediatr-us, v144, p93, doi 10.1016/s0022-3476(03)00449-9 | 107 | 693 |
| quartier p, 1999, j pediatr-us, v134, p589, doi 10.1016/s0022-3476(99)70246-5 | 109 | 680 |
| dupuis s, 2003, nat genet, v33, p388, doi 10.1038/ng1097 | 112 | 668 |
| orange js, 2012, j allergy clin immun, v130, ps1, doi 10.1016/j.jaci.2012.07.002 | 128 | 663 |
| chapel hm, 2000, j clin immunol, v20, p94, doi 10.1023/a:1006678312925 | 121 | 662 |
| aydin se, 2015, j clin immunol, v35, p189, doi 10.1007/s10875-014-0126-0 | 107 | 644 |
| al-herz w, 2011, front immunol, v2, doi 10.3389/fimmu.2011.00054 | 156 | 642 |
| rigaud s, 2006, nature, v444, p110, doi 10.1038/nature05257 | 136 | 640 |
| ochs hd, 2006, j clin immunol, v26, p265, doi 10.1007/s10875-006-9021-7 | 128 | 634 |
| buckley rh, 1999, new engl j med, v340, p508, doi 10.1056/nejm199902183400703 | 137 | 627 |
| güngör t, 2014, lancet, v383, p436, doi [10.1016/s0140-6736(13)62069-3 10.1016/s0140-6736(13)62069-3] | 129 | 627 |
| richards s, 2015, genet med, v17, p405, doi 10.1038/gim.2015.30 | 200 | 621 |
| van den berg jm, 2009, plos one, v4, doi 10.1371/journal.pone.0005234 | 173 | 618 |
| hammarström l, 2000, clin exp immunol, v120, p225, doi 10.1046/j.1365-2249.2000.01131.x | 106 | 617 |
| glocker eo, 2009, new engl j med, v361, p1727, doi 10.1056/nejmoa0810719 | 105 | 613 |
| noguchi m, 1993, cell, v73, p147, doi 10.1016/0092-8674(93)90167-o | 117 | 599 |
| antoine c, 2003, lancet, v361, p553, doi 10.1016/s0140-6736(03)12513-5 | 151 | 595 |
| revy p, 2000, cell, v102, p565, doi 10.1016/s0092-8674(00)00079-9 | 104 | 593 |
| döffinger r, 2001, nat genet, v27, p277, doi 10.1038/85837 | 108 | 589 |
| notarangelo ld, 2010, j allergy clin immun, v125, ps182, doi 10.1016/j.jaci.2009.07.053 | 130 | 582 |
| cavazzana-calvo m, 2000, science, v288, p669, doi 10.1126/science.288.5466.669 | 113 | 567 |
| wood p, 2007, clin exp immunol, v149, p410, doi 10.1111/j.1365-2249.2007.03432.x | 103 | 562 |
| kuhns db, 2010, new engl j med, v363, p2600, doi 10.1056/nejmoa1007097 | 118 | 559 |
| gathmann b, 2009, clin exp immunol, v157, p3, doi 10.1111/j.1365-2249.2009.03954.x | 105 | 557 |
| moratto d, 2011, blood, v118, p1675, doi 10.1182/blood-2010-11-319376 | 104 | 555 |
| gardulf a, 2006, j clin immunol, v26, p177, doi 10.1007/s10875-006-9002-x | 104 | 551 |
| gatti ra, 1968, lancet, v2, p1366 | 101 | 551 |
| aiuti a, 2002, science, v296, p2410, doi 10.1126/science.1070104 | 109 | 548 |
| aiuti a, 2009, new engl j med, v360, p447, doi 10.1056/nejmoa0805817 | 108 | 546 |
| gardulf a, 2004, j allergy clin immun, v114, p936, doi 10.1016/j.jaci.2004.06.053 | 133 | 543 |
| perez ee, 2017, j allergy clin immun, v139, ps1, doi 10.1016/j.jaci.2016.09.023 | 123 | 542 |
| coffey aj, 1998, nat genet, v20, p129, doi 10.1038/2424 | 111 | 529 |
| bousfiha a, 2022, j clin immunol, v42, p1508, doi 10.1007/s10875-022-01352-z | 147 | 523 |
| picard c, 2003, science, v299, p2076, doi 10.1126/science.1081902 | 117 | 513 |
| tangye sg, 2021, j clin immunol, v41, p666, doi 10.1007/s10875-021-00980-1 | 112 | 512 |
| notarangelo ld, 2020, sci immunol, v5, doi 10.1126/sciimmunol.abb1662 | 107 | 511 |
| bustamante j, 2014, semin immunol, v26, p454, doi 10.1016/j.smim.2014.09.008 | 126 | 509 |
| glocker eo, 2009, new engl j med, v361, p2033, doi 10.1056/nejmoa0907206 | 103 | 497 |
| segal bh, 2000, medicine, v79, p170, doi 10.1097/00005792-200005000-00004 | 136 | 491 |
| chan k, 2005, j allergy clin immun, v115, p391, doi 10.1016/j.jaci.2004.10.012 | 111 | 476 |
| ott mg, 2006, nat med, v12, p401, doi 10.1038/nm1393 | 104 | 457 |
| henter ji, 2007, pediatr blood cancer, v48, p124, doi 10.1002/pbc.21039 | 127 | 453 |
| sayos j, 1998, nature, v395, p462, doi 10.1038/26683 | 100 | 438 |
| ochs hd, 2006, j allergy clin immun, v117, p725, doi 10.1016/j.jaci.2006.02.005 | 103 | 437 |
| schmid jp, 2011, blood, v117, p1522, doi 10.1182/blood-2010-07-298372 | 102 | 421 |
| derry jmj, 1994, cell, v78, p635, doi 10.1016/0092-8674(94)90528-2 | 108 | 376 |
| zhang q, 2020, science, v370, doi 10.1126/science.abd4570 | 104 | 313 |
| meyts i, 2021, j allergy clin immun, v147, p520, doi 10.1016/j.jaci.2020.09.010 | 116 | 263 |
| shearer wt, 2003, j allergy clin immun, v112, p973, doi 10.1016/j.jaci.2003.07.003 | 109 | 252 |

**Figure S18. Co-citation network analysis of references**

**
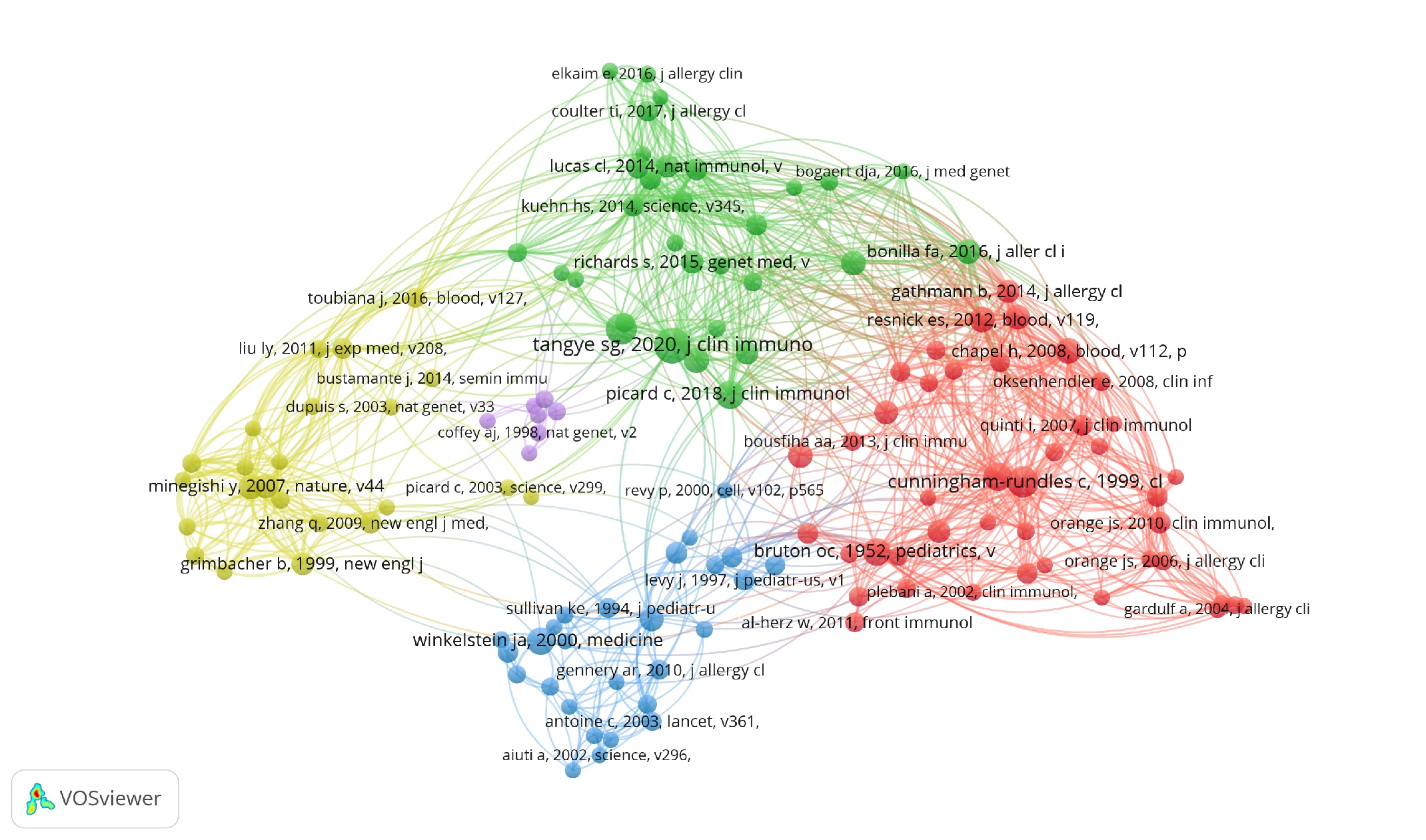
**

**Figure S19. The 100 most frequent keywords**


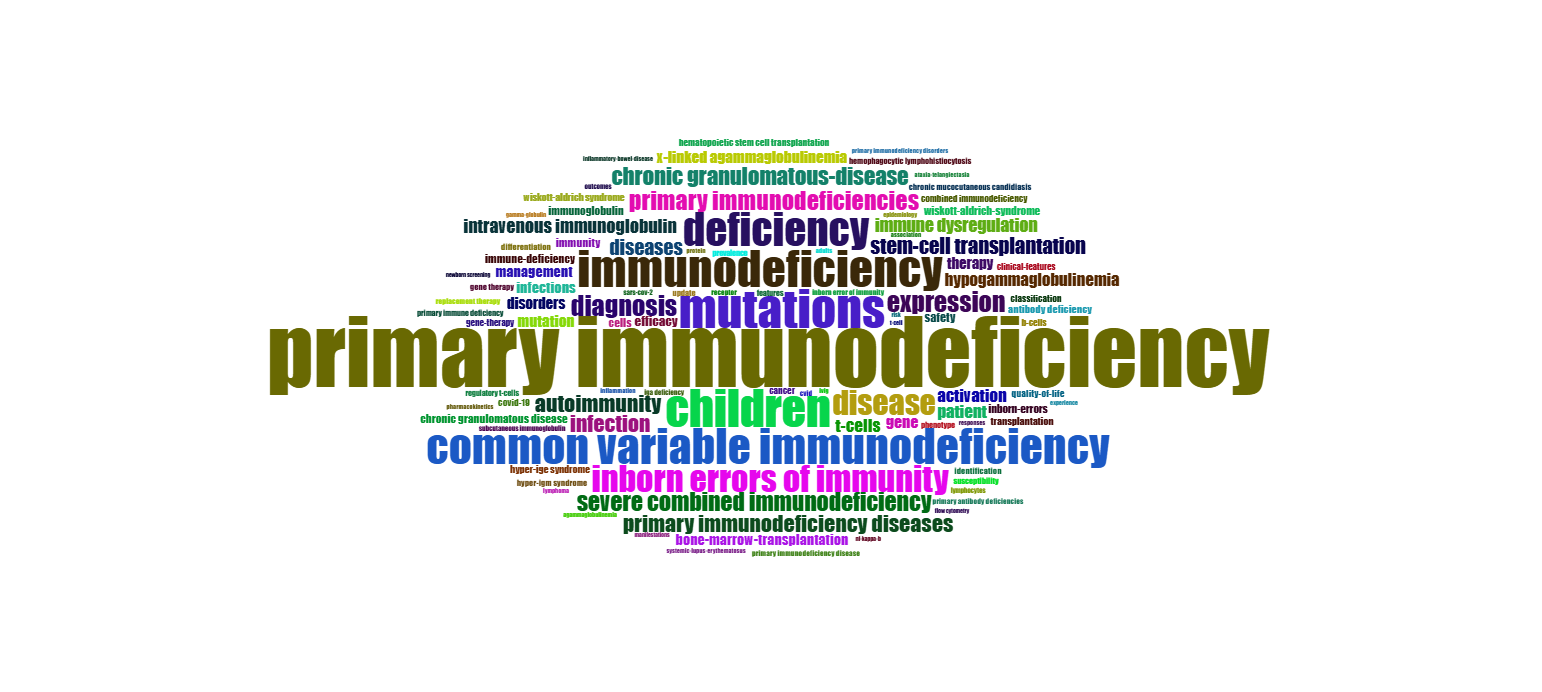


**Figure S20. The trend of keywords for topics**


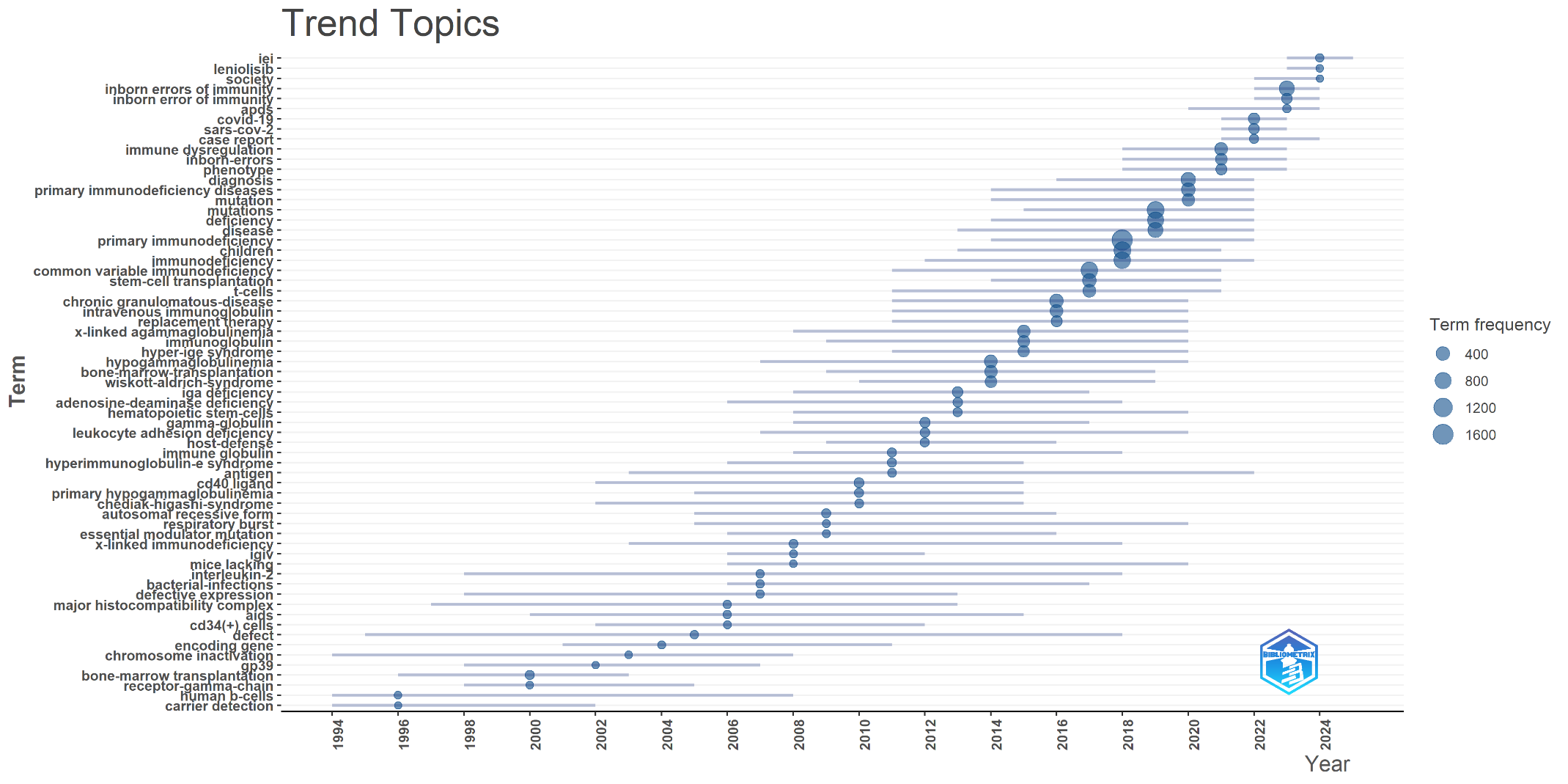


**Figure S21. Top 50 keywords with the strongest citation bursts**


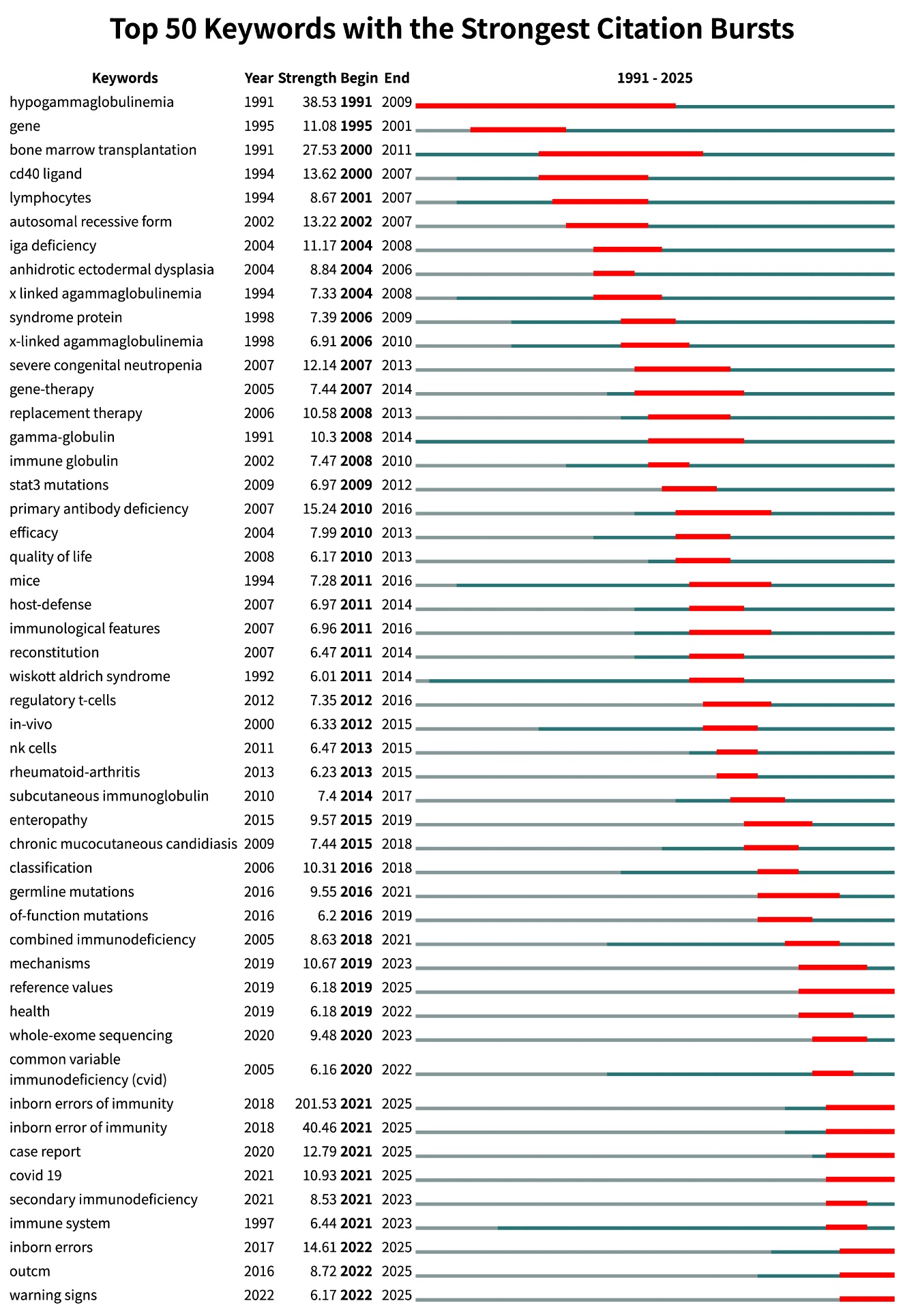


**Table S4. Regional comparison of IEI research, challenges, and recommended actions**

| **Region** | **Research Landscape (Keywords)** | **Key Challenges** | **Policy & Action Recommendations** |
| --- | --- | --- | --- |
| North America & Western Europe | Leaders (High output, Frontier research) | High costs, Access disparities | Develop reimbursement models, Establish long-term registries |
| East Asia | Rising Contributors (Rapid growth, Increasing collaboration) | Insufficient local data, Incomplete screening coverage | Map local genetic landscape, Expand newborn screening (NBS) |
| Latin America & Middle East | Emerging Hubs (Active regional networks, Limited output) | Diagnostic bottlenecks, Poor access to therapies | Improve access to genetic testing, Secure essential treatments |
| Sub-Saharan Africa & Southeast Asia | Data-Scarce Regions (Data scarcity, Insufficient collaboration) | Lack of infrastructure, Severe workforce shortage | Build foundational systems (diagnostics, registries), Urgent capacity building |
